# Emergence of unprecedented climate change in projected future precipitation

**DOI:** 10.1038/s41598-020-61792-8

**Published:** 2020-03-16

**Authors:** Shoji Kusunoki, Tomoaki Ose, Masahiro Hosaka

**Affiliations:** 1 Department of Atmosphere, Ocean and Earth System Modeling Research, Meteorological Research Institute, Tsukuba, Ibaraki Japan; 2 Faculty of Societal Safety Sciences, Kansai University, Takatsuki, Osaka Japan; 3 Department of Climate and Environment Research, Meteorological Research Institute, Tsukuba, Ibaraki Japan

**Keywords:** Atmospheric dynamics, Projection and prediction, Atmospheric dynamics

## Abstract

The future time of emergence when precipitation changes due to anthropogenic influences begins to continuously exceed the previous maximum value is defined as the ‘tipping year’ Historical experiments and future experiments simulated by state-of-the-art climate models were utilized. A total of 510,000 time series from year 1856 to 2095 were generated by sampling the natural internal variability in precipitation. The time evolutions of internal variability in the whole time period were estimated from the combination of past and future experiments with preindustrial control experiments. A large ensemble size enabled an estimation of the probability density function of the tipping year at each grid point, providing precise information on the uncertainty of the projection. The tipping year of average precipitation emerges earlier in high latitudes than in lower latitudes. In some regions in lower latitudes and mid-latitudes, the tipping year of intense precipitation emerges faster than that of average precipitation. The tipping years of average and intense precipitation are earlier for higher anthropogenic forcing scenarios than for lower scenarios. The global average of the tipping year for intense precipitation might be attributed to the enhancement of the thermodynamic effect (moisture) rather than the dynamic effect (vertical motion).

## Introduction

In the context of climate change, a ‘tipping point’ is defined as a threshold of abrupt and irreversible change^1^. The increase in temperature caused by global warming will lead to the crossing of various tipping points that will increase risks across various sectors and regions in terms of both natural ecosystems and human society^2–5^. However, the projections of future climate by climate models contain intrinsic uncertainties that originate from factors such as emissions scenarios of greenhouse gases (GHGs) and other forcing agents, model structure and parameterization, and internal natural variability^6,7^. The Earth’s climate system contains inevitable chaotic nonlinearity that gives rise to unpredictable internal natural variability. In particular, the variability associated with precipitation is much greater than that associated with temperature. Future changes in precipitation might lead to nonlinear local temperature changes which would cause substantial variability in temperature^8^. The relative magnitude of the forced response (signal) to internal natural variability (noise), known as the signal-to-noise ratio, can be used to quantify the reliability of projections^9^. Due to the high noise levels associated with the simulation of precipitation, the tipping point of precipitation change and its dependence on the magnitude of anthropogenic forcing has yet to be fully investigated. The purpose of this paper is to extract a meaningful signal for the tipping point of precipitation from the large amount of unpredictable noise and test the reliability of this signal. We also intend to answer the question of whether in comparison to lower forcing, larger anthropogenic external forcing accelerates the emergence of the tipping point.

## Tipping year

Daily precipitation simulated by the Atmosphere-Ocean General Circulation Models (AOGCMs) and the Earth System Models (ESMs) that participated in the fifth phase of the Coupled Model Intercomparison Project (CMIP5)^10^ were utilized for this study (Tables S1 and S2). CMIP5 (Table S2) models perform better than previous generation models of the third phase of the Coupled Model Intercomparison Project (CMIP3, Table S3) in simulating precipitation (See Methods, Figs. S1–S3). The annual mean precipitation *pav* and the indices of extreme precipitation events such as the annual maximum of 5-day precipitation *r5d* and the annual maximum of daily precipitation *r1d* were calculated from a preindustrial control experiment ‘piControl’, a historical experiment ‘historical’, and a future projection experiment assuming the Representative Concentration Pathways (RCP)^9,11^ scenarios designated by the CMIP5 protocol.

**Figure 1:**
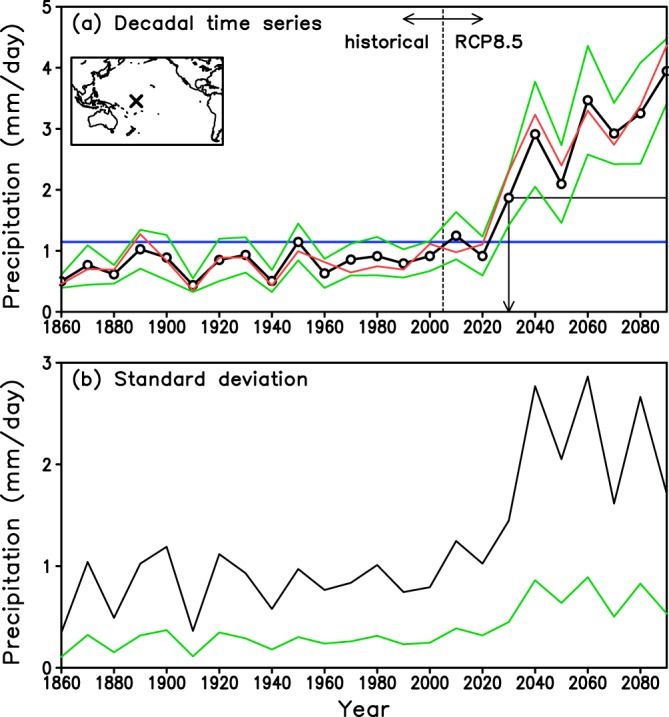
Method used to define the tipping year. (**a**) The black line with open circle shows the time series of the decadal average of annual mean precipitation *pav* simulated by the model MRI-CGCM3 at the grid point (179.5°W, 0.5°N; X mark in the inset map). Here, for example, the data point for the year 1860 is used as the 10-year average over the period 1856–1865. Data for the period 1860–2000 were derived from the historical simulation. Data for the period 2010–2090 were derived from the RCP8.5 simulation. The blue horizontal line denotes the maximum value of 1.15 mm day^−1^ from the historical simulation for the year 1950. The black horizontal line denotes the value of 1.87 mm day^−1^ from the tipping year 2030. The two green lines show the range of decadal natural variability *V*_*d*_ (one standard deviation). The red line is an example of a randomized time series generated using the Monte Carlo method within the range of decadal variability (green lines). (**b**) Year-to-year natural variability *V*_*y*_ (black) and decadal variability *V*_*d*_ (green) of *pav*. The decadal variability was obtained using the ratio of year-to-year variability to decadal variability estimated from the piControl experiment (Fig. S10c).

Figure 1 illustrates the method to determine the ‘tipping year’ which is defined as the start of the period in which precipitation consistently exceeds the maximum value obtained from the historical experiment^4^. The black line in Fig. 1a is an example of the temporal evolution of the decadal average of *pav* in a historical experiment and an RCP8.5 experiment simulated by a specific AOGCM at a grid point in the central tropical Pacific. The data point for the year 1860 is taken to represent the 10-year average for the period 1856–1865. After year 2030 (2026–2035), precipitation continuously exceeds the maximum value of the historical experiment. Thus, the tipping year is 2030 (2026–2035) in this case. However, if we had extended the target period to years beyond 2100, precipitation might decrease below the value in 2030 (2026–2035)^12^. Therefore, 2030 (2026–2035) is not the beginning of ‘irreversible’ change from the perspective of longer time period beyond 2100.

The black line in Fig. 1b is the year-to-year natural variability evaluated from the standard deviation for each decade. The green line in Fig. 1b is the natural variability of the decadal average of *pav* estimated from a piControl experiment. The two green lines in Fig. 1a show the range of decadal variability, which increases after 2030 owing to the increases in year-to-year natural variability. The red line in Fig. 1a is an example of one of the randomized time series distributed within the range of decadal variability using the Monte Carlo method.

## Probability density function

**Figure 2:**
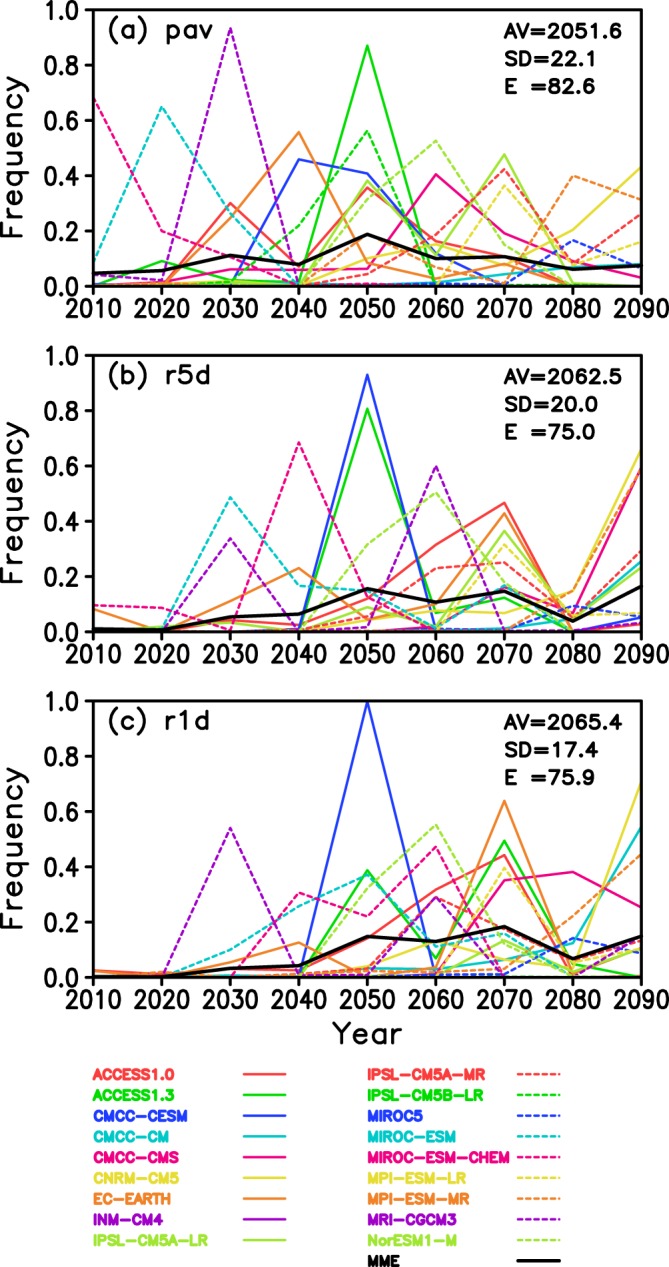
Probability Density Function (PDF) of the tipping year for RCP8.5. (**a**) The PDFs for *pav* were calculated using the 10,000 randomized time series for each model at the grid point (179.5°W, 0.5°N marked with × in the inset map of Fig. 1a). For the individual model specifications, see Table S2. As the tipping year is missing in some of the time series, the integration of each PDF can be less than unity. The black thick line is the Multi-Model Ensemble (MME) average for which the root mean square errors of the modelled global distribution of *pav* (Fig. S1a) are used as weighting factors (Text S1) in the averaging. The value of AV, SD and E in the panel denote the expectation value *TY*_*av*_ (year), standard deviation *TY*_*sd*_ (year) and existent rate *E* (%) of MME, respectively. *E* is identical to the integral of PDF of MME. (**b**) *r5d*. (**c**) *r1d*.

Figure 2a is an example of the probability density functions (PDFs) of *pav* derived from 18 models at a grid point in the central tropical Pacific for the RCP8.5 scenario. Note that in some cases the tipping year does not exist, so that the integral of the PDF can be less than 1. The black line shows the multi-model ensemble (MME) average with a weighting factor that takes into account the reproducibility of the present-day climatology (Figs. S1–S3, Text S1). The expected value of the PDF of the MME average *TY*_*av*_ is 2051.6, which is the most plausible projection of the tipping year at this grid point. Here, we propose two kinds of reliability information. One is the standard deviation of the PDF of the MME *TY*_*sd*_, which measures the spread of the PDF distribution. A smaller *TY*_*sd*_ indicates relatively higher reliability for the projected tipping year. In the case of Fig. 2a, the value of *TY*_*sd*_ is 22.1 years. The other reliability measure is the existing rate of tipping year *E* (%) among 10,000 samples. A total of 180,000 (18 models × 10,000 randomized samples) time series were generated, but the tipping year is missing in some of them where the future increase in precipitation is small. In Fig. 2a, *E* is 82.6%. A larger *E* indicates higher reliability for the projected tipping year. In terms of the MME average (black thick line), tipping years (*TY*_*av*_) of *r5d* (Fig. 2b) and r1d (Fig. 2c) are later than those of *pav* (Fig. 2a). The large spread of the tipping years among the climate models in Fig. 2 is consistent with those of previous studies^4,5^.

## Geographical distribution of tipping year

**Figure 3:**
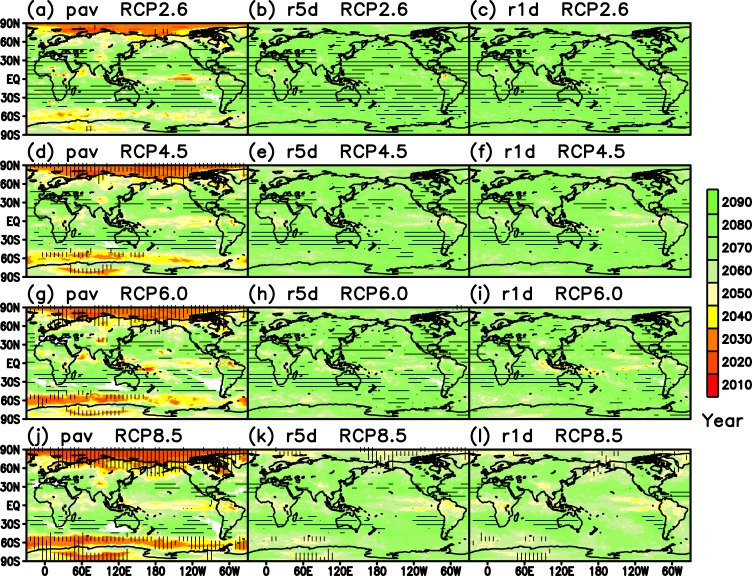
Distributions of tipping year. Color shading shows the expectation value of the PDF of the MME average *TY*_*av*_. The horizontal hatching denotes the standard deviation of the PDF *TY*_*sd*_ less than 10 year. The vertical hatching denotes that the existence rate of the tipping year *E* exceeds 90%. The white regions indicate a missing of tipping year. (First column: **a,d,g,j**) *pav*. (Second column: **b,e,h,k**) *r5d*. (Third column: **c,f,i,l**) *r1d*. (First row: **a** to **c**) RCP2.6. (Second row: **d** to **f**) RCP4.5. (Third row: **g** to **i**) RCP6.0. (Fourth row: **j** to **l**) RCP8.5.

Figure 3 shows the distribution of *TY*_*av*_ for three precipitation indices and four RCP scenarios based on the PDF of the MME average. The degree of reliability is displayed by horizontal (*TY*_*sd*_) and vertical (*E*) hatching. In the case of *pav* for RCP2.6 (Fig. 3a), the tipping year is earlier at the high latitudes, especially over the Arctic region. The tipping year at the midlatitudes and in the tropics is later than that at the high latitudes. In some of the subtropical regions that correspond to dry areas under the present-day climate, the tipping year is missing (white), especially in the Southern Hemisphere. Many regions at latitudes of approximately 30 degrees are covered with horizontal hatching (small *TY*_*sd*_), indicating relatively higher reliability compared with that of other regions.

In the case of *r5d* for RCP2.6 (Fig. 3b), the tipping year is generally later than that of *pav* (Fig. 3a), but the tipping year exists over the whole world, including the subtropics, where the tipping year is missing for *pav* (Fig. 3a) in some regions. As to *r1d* for RCP2.6 (Fig. 3c), the pattern of tipping is almost similar to that of *r5d* (Fig. 3b). In the case of the higher forcing scenarios of RCP4.5 (Fig. 3d–f) and RCP 6.0 (Fig. 3g–i), the tipping year tends to be faster than those of RCP2.6 (Fig. 3a–c). In the highest scenario of RCP8.5 (Fig. 3j–l), the spatial distributions of tipping year are almost similar to those of the lower scenarios, but the tipping year occurs more rapidly than that of the other scenarios.

**Figure 4:**
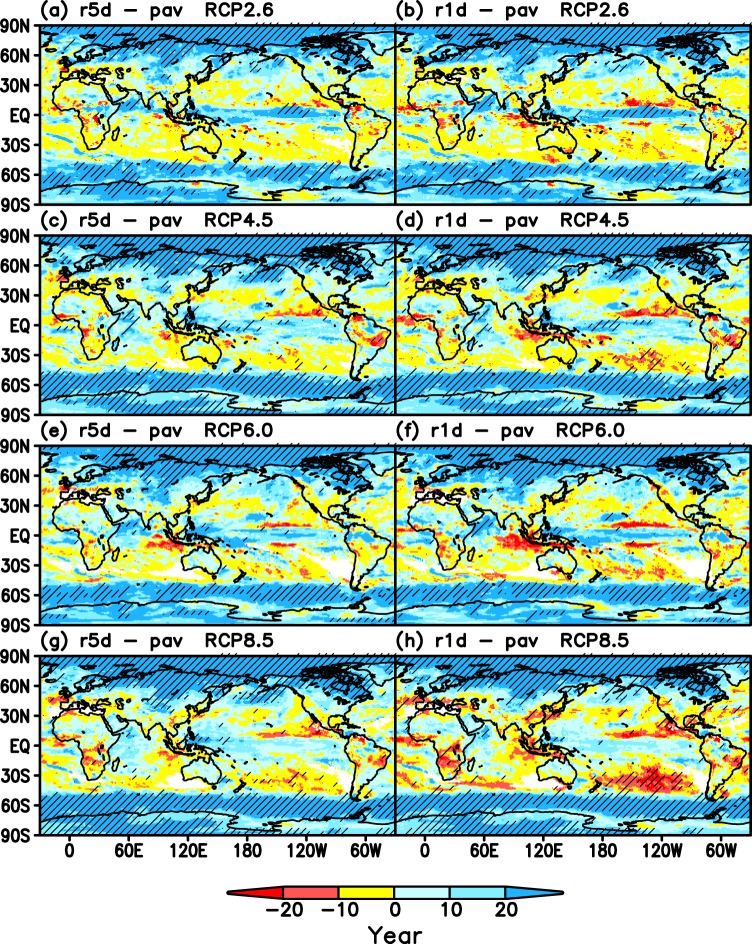
Differences of tipping year. Hatched region indicates a significance level exceeding 95% based on Student’s t-test (See Text S2 for detail). (**a**) *r5d* - *pav* for RCP2.6. (**b**) *r1d* - *pav* for RCP2.6. (**c**,**d**) Same as (**a**,**b)** but for RCP4.5. (**e**,**f**) Same as **a** and **b** but for RCP6.0. (**g**,**h**) Same as **a**,**b** but for RCP8.5.

Figure 4 displays the differences in tipping year as *r5d* - *pav* and *r1d* - *pav*. In the case of *r5d* for RCP2.6 (Fig. 4a), the tipping year of *r5d* is faster than that of *pav* in some regions (red) in the tropics and subtropics. The red region area for *r1d* (Fig. 4b) is larger than that for *r5d* (Fig. 4a). The red region area for the higher emissions scenarios (Fig. 4c–h) are larger than those of RCP2.6 (Fig. 4a,b). In paricular, in the case of *r1d* for RCP8.5 (Fig. 4h), some red regions extend further to the midlatitudes. This suggests that the tipping year of the most intense precipitation appears faster than that of moderate and average precipitation in some regions over the tropics, subtropics and midlatitudes for the highest emissions scenario RCP8.5.

Cumulative probabilities integrated from 2010 to 2050 (2006–2055) are calculated on a grid point base to estimate the possibility of emergence of tipping years (Text S3, Fig. S4). The dependency of the difference in cumulative probability on precipitation indices and forcing scenario (Fig. S5) is almost similar and consistent with that of tipping year (Fig. 4).

Analyses based on the expectation value (Figs. 3 and 4) and cumulative probability (Figs. S4 and S5) both indicate that the possibility of the emergence of tipping years for intense precipitation will be larger than that for moderate and average precipitation in some regions of the tropics, subtropics and midlatitudes. If we focus on the highest RCP8.5 scenario, then the earlier emergence of the tipping year of *r1d* compared to that of *pav* consistently appears both in Figs. 4h and S5h over many regions such as the Mediterranean, the equatorial region in Western Africa, at approximately 30°S in Africa, East Asia, the Maritime continent, the eastern tropical Pacific Ocean, the Amazon and approximately 30°S in the South Pacific Ocean.

## Discussion

The tipping year is essentially determined by the relative magnitude of precipitation change and decadal natural variability. The latitudinal profile of the tipping year (Fig. S6w) is strongly correlated to the profile of the ratio of precipitation change to decadal natural variability (Fig. S6d) rather than precipitation change (Fig. S6b). In Fig. S6b *pav* decreases at approximately 20°N and 10–30°S, leading to the absence of tipping years in some areas at this latitude (Fig. 3j). On the other hand, *r1d* increases over all latitudes (Fig. S7b), leading to the emergence of tipping years over the whole globe (Fig. 3l). This result is consistent with previous findings that the increase in intense precipitation globally is much larger, even in the subtropics^13–17^.

As the natural variability in precipitation is much larger than that of the surface air temperature, a decadal average was used to enhance the signal-to-noise ratio, which is defined as the relative magnitude of the forced precipitation response (signal) to the natural variability (noise). Averaging over a longer time reduces noise, but the temporal resolution is also reduced. Conversely, averaging over a shorter period increases noise, but the temporal resolution also increases. Figure S8 illustrates the dependency of the tipping year on the time scale of the averaging period. If we use annual precipitation (Fig. S8a), then the tipping year appears at the end of the 21st century over only the high latitudes because of the larger natural variability. If we increase the length of the averaging period, then the area of emergence of the tipping year becomes larger and the tipping year appears earlier because of the smaller natural variability (Fig. S8b–j). The dependency of the tipping year on the length of the averaging period ise clearly illustrated in terms of the zonal average (Fig. S9). Consequently, the decadal average was selected to balance this trade-off between the length of the averaging period and the temporal resolution (Figs. S8 and S9). Moreover, 10-year averages might be much easier to understand than 9-year or 11-year averages, because we are using the decimal counting system for year.

A possible alternative approach to the decadal average is the 10-year running mean. The spatial distribution of the tipping year derived from the decadal average and 10-year running mean is almost similar for *pav* (Fig. S10a,d), *r5d* (Fig. S10b,e) and *r1d* (Fig. S10c,f). Consequently, differences between tipping years derived from decadal averages and 10-year running means are generally within approximately 10 years except for northern high latitudes and Antarctica (Fig. S10g–i).

The dependency of the tipping year on the magnitude of the RCP scenario was investigated from a global perspective. Figure S11 depicts the relation between the global average of the tipping year and the magnitude of radiative forcing (RF) of the RCP scenarios. In the case of *pav* (Fig. S11b), the tipping year is almost linearly related to RF with an inclination of −1.61 year (w m^−2^)^−1^. A similar linear relation also holds for *r5d* (Fig. S11b) and *r1d* (Fig. S11c) with larger inclinations. This suggests that the tipping year of intense precipitation is much more sensitive to RF than that of moderate and average precipitation.

The number of models used in each RCP simulation is different (Table S1). Thus, the calculation of Fig. S11 should be based on the same models for all four scenarios to accurately evaluate the dependency on RF. Therefore, the same calculation was repeated using 7 models that conducted the future simulations for all four scenarios (Fig. S12). Nevertheless, the results shown in Fig. S12 are similar to those shown in Fig. S11.

The climate sensitivity of a model is defined as the change in the global average surface air temperature at the doubling of CO_2_ concentration^18^. The PDFs in Fig. 2 show large dispersion that originates fundamentally from the large differences in climate sensitivity among the CMIP5 models^18^. Models with high climate sensitivity generate large warming, which leads to increased water vapor in the atmosphere and, hence a larger increase in precipitation^19^. As a consequence, models with high climate sensitivity tend to project an earlier tipping year (Fig. S13). The results confirmed the high statistical significance of the relationship between the climate sensitivity of the models and the tipping year, although climate sensitivities were available only for ten of the models used in this study (Table S4).

Since a dynamic effect also contributes to future precipitation changes as well as thermodynamic effects, we decomposed *r1d* changes into thermodynamic contributions (moisture) and dynamic contributions (vertical motion) for RCP8.5 (Text S4, Fig. S14)^20^. We found that the globally averaged tipping year of *r1d* is well correlated with the thermodynamic effect rather than the dynamic effect (Fig. S15). This indicates that the tipping year of intense precipitation is affected by changes in moisture rather than changes in vertical motion.

## Methods

### Models and experiments

Simulated precipitation data generated by AOGCMs and ESMs that participated in CMIP5^10^ were used for this study. The data were obtained from https://pcmdi.llnl.gov/mips/cmip5/. The annual mean precipitation *pav*, the annual maximum of 5-day precipitation *r5d* and the annual maximum of daily precipitation *r1d* were calculated from daily precipitation in a preindustrial control experiment ‘piControl’, a historical experiment ‘historical’, and a future experiment ‘RCP8.5’^9,11^. The global mean surface temperature will increase by as much as 4.8 °C by the end of the 21st century relative to the preindustrial period. Fourteen AOGCMs and four ESMs (Tables S1 and S2) were selected that simulated a period exceeding 250 years for the piControl experiment, 150 years (1856–2005) for the historical experiment and 90 years (2006–2095) for the RCP8.5 experiment. The piControl experiments were used to evaluate the natural internal variability in precipitation. The selected CMIP5 (Tables S1 and S2) models are state-of-the-art models that perform better than, or as well as, CMIP3 (Table S3) models in simulating the global distribution of *pav* (Fig. S1), *r5d* (Fig. S2) and *r1d* (Fig. S3). To investigate the dependency of the forcing scenario on future precipitation changes, we also used the daily precipitation data from RCP2.6, RCP4.5 and RCP6.0, although the number of models is limited compared with the number of models in RCP8.5 (Table S1).

### Tipping year

The black line with an open circle in Fig. 1a is an example of the temporal evolution of *pav* simulated by the MRI-CGCM3 model at a grid point in the central tropical Pacific for the historical and RCP8.5 experiments. The decadal (10 year) average of *pav* is used to reduce the natural variability of precipitation and enhance the signal-to-noise level of future precipitation change. For example, the data point for the year 1860 is taken to represent the 10-year average for the period 1856–1865. In Fig. 1a, the simulated precipitation from the historical experiment is relatively stationary, with a maximum *P*_*max*_ of 1.15 mm day^−1^ in 1950. In contrast, precipitation is projected to increase in the RCP8.5 experiment. Precipitation exceeds *P*_*max*_ in 2010 (2006–2015) with a value of 1.25 mm day^−1^, but will fall below *P*_*max*_ in 2020 (2016–2025). Then, precipitation exceeds *P*_*max*_ in 2030 (2026–2035) with a value of 1.87 mm day^−1^. Thereafter precipitation continuously exceeds *P*_*max*_ until the end of the 21st century. The ‘tipping year’ is defined as the start of the period in which precipitation consistently exceeds the maximum value obtained from the historical experiment^4^. In the case of Fig. 1a, the tipping year is 2030 (2026–2035).

### Grid system

At the original grids of each model, precipitation indices of *pav, r5d* and *r1d* are calculated for each year. After taking a 10-year average, all the model data are bilinear interpolated onto a common 1-degree regular grid where the model performance (Figs. S1–3) and the weights for the MME average (Text S1) are evaluated.

### Natural internal variability

To estimate the natural internal variability of the precipitation originating from the chaotic nature of the climate system, we used piControl experiments in which the external forcings were fixed at the preindustrial level (Table S1, Fourth column). Figure S16 shows an example of a time series of *pav* from the central tropical Pacific simulated in the piControl experiment by the MRI-CGCM3 model. Precipitation shows large year-to-year variability, as well as decadal or longer-period variability but with no statistically significant trend. The main purpose of the piControl experiment was to confirm that the model showed no significant climate drift, which would have distorted the historical and future experiments and reduced the reliability of the projected future changes^21^. It was confirmed that the models used in this study were generally free of strong trends (Fig. S17). To ensure the complete elimination of climate drift, linear trends were removed from the original time series at each grid point in each model experiment. The whole detrended time series was divided into contiguous 10-year periods and the average and standard deviations were calculated for each period. The year-to-year natural variability *S*_*y*_ (Fig. S18) was evaluated using the average of all standard deviations. The decadal natural variability *S*_*d*_ (Fig. S19) was evaluated using the standard deviation of all of the decadal averages. Most models reproduced a maximum *S*_*y*_ in the tropical regions of the Pacific Ocean, although the models tend to overestimate variability over the Indian Ocean (Fig. S18). Most models reproduced the maximum *S*_*d*_ in the tropical regions of the Pacific Ocean, although some models tend to underestimate variability (Fig. S19).

### Time-dependent decadal variability

In the historical and RCP8.5 experiments, the year-to-year natural variability *V*_*y*_ of the models was evaluated from the standard deviation for each decade. In association with the increase in precipitation after 2030 (Fig. 1a, black line with open circle), *V*_*y*_ is also projected to increase in the future (Fig. 1b, black line). The decadal natural variability *V*_*d*_ of the models at each grid point was estimated as *V*_*y*_ × *r* (Fig. 1b, green line) where *r* = *S*_*y*_/*S*_*d*_ is the ratio of decadal variability to year-to-year variability derived from the piControl experiment (Fig. S20c). The distribution of *r* is nearly uniform, with a constant value of approximately 30% (Fig. S20c). Next, the range of decadal variability (Fig. 1a, green lines) of the decadal average of *pav* in the historical and RCP8.5 experiments was specified by adding and subtracting *V*_*d*_/2 from the original time series (Fig. 1a, black line with open circles). The range of decadal variability (Fig. 1a, green lines) also increases after 2030 owing to the increases in *V*_*y*_ and *V*_*d*_ (Fig. 1b).

### Sensitivity to the magnitude of natural variability

Tipping year is influenced by the range of natural variability of decadal average precipitation. Figure S21 illustrates the sensitivity of the tipping year on the magnitude of decadal natural variability. Larger variability (orange, red lines) leads to the larger spread of PDF distribution as is naturally expected from the increase in extreme values included in the sampling of decadal average precipitation.

### Large ensemble simulations

To evaluate the uncertainty associated with the tipping year caused by natural variability, the Monte Carlo method was used to generate large ensembles of randomized time series spanning the range of decadal natural variability (green lines in Fig. 1b). The red line in Fig. 1b is an example of one of the generated time series. A total of 10,000 randomized time series were created at each grid point for each model. Then, the tipping year was calculated for each time series. The large ensemble size enabled us to directly estimate the probability density function (PDF) or frequency distribution of the tipping year at every grid point for each model. For the RCP2.6, 4.5 and 6.0 experiments, we followed similar procedures as those in the RCP8.5 experiment.

### Number and length of experiments

In this study, the total number of simulated years by climate models was 15,190 years (Table S1, Total years). Since the number of RCP experiments is 51 = 10 models (RCP2.6) + 16 (RCP4.5) + 7 (RCP6.0) + 18 (RCP8.5), the total number of randomized time series is 510,000 = 51 × 10,000. The length of each time series is 240 years, which consists of 150-year (1856–2005) historical experiments and 90-year (2006–2095) future RCP experiments. Thus, the total length of the randomized time series is 122,400,000 years = 510,000 time series x 240 years.

The data sources used in this study are listed in Table S5.

## Supplementary information

Supplement.
