# Author Correction: Emergence of unprecedented climate change in projected future precipitation

**DOI:** 10.1038/s41598-020-63945-1

**Published:** 2020-04-29

**Authors:** Shoji Kusunoki, Tomoaki Ose, Masahiro Hosaka

**Affiliations:** 1 Department of Atmosphere, Ocean and Earth System Modeling Research, Meteorological Research Institute, Tsukuba, Ibaraki Japan; 2 Faculty of Societal Safety Sciences, Kansai University, Takatsuki, Osaka Japan; 3 Department of Climate and Environment Research, Meteorological Research Institute, Tsukuba, Ibaraki Japan

Correction to: *Scientific Reports* 10.1038/s41598-020-61792-8, published online 16 March 2020

The Supplementary Information file originally published with this Article contained and error in the footnote for Table S4, where

“Out of 18 models used in this study, climate sensitivities of 10 models are only available from the Table 9.5 in (11). For technical detail, see (11).”

now reads:

“Out of 18 models used in this study, climate sensitivities of 10 models are only available from the Table 9.5 in Flato *et al.* (2013). For technical detail, see Flato *et al.* (2013).”

This error has been corrected in the Supplementary Information that accompanies the Article.

