# Supplementary material for: Emergence of unprecedented climate change in projected future precipitation
Source: Sci Rep. 2020 Mar 16;10:4802. doi: 10.1038/s41598-020-61792-8 (PMC7075997; doi:10.1038/s41598-020-61792-8)
Supplement: Supplementary file 1 — Supplement. [file 41598_2020_61792_MOESM1_ESM.pdf]

Supplementary information

Title: Emergence of unprecedented climate change in projected future precipitation
Author: Shoji Kusunoki, Tomoaki Ose, Masahiro Hosaka
Journal name: Scientific Reports

Text S1 to S4
Figures S1 to S21
Tables S1 to S5
References

Text S1. Weights for the Multi-Model Ensemble (MME) average.

For the calculation of the MME average, we took into consideration weighting factors that reflect the reproducibility of present-day climatology. In case of *pave*, we used Root Mean Square Errors (RMSE) of global precipitation distribution (Fig. S1a). We defined weight w as

$$w(x) = \frac{1}{(ax^2 + 1)} \quad (1)$$

where $x = \frac{rms}{R_{OBS}}$,

rms: RMSE

R_{OBS} : Global average of observed *pav*,

a : Sensitivity parameter determining the shape and slope of the curve $w(x)$.

w becomes unity if x is zero, while w becomes zero if x goes to infinity. We set $a = 1$ so that w is most sensitive to x around $x = 0.6$.

We have also tested spatial correlation coefficient *cor* (Fig. S1b) to define weight w as

$$w(x) = \frac{cor + 1}{2}. \quad (2)$$

w becomes unity if *cor* is 1, while w becomes zero if *cor* is -1. We found that tipping year and related statistics of the MME average by equation (2) is almost similar to that by (1). Therefore, in this paper we showed the results based on equation (1). We apply this method to *r5d* and *r1d* using RMSE in Figs. S2 and S3, respectively

Text S2. Statistical significant test in Fig. 4.

We used the Student's t-test to evaluate the statistical significance of differences between *r5d* and *pav*, *r1d* and *pav*. Let the number of simulations which show the emergence of tipping year at a certain grid point for *pav* and that for *r5d* denoted by N_{pav} and N_{r5d}, respectively. In case of Fig. 4a at the grid point near Singapore (103.5°E, 1.5°N), N_{pav} = 9,690 and N_{r5d} = 26,076. The degree of freedom (DOF) at this grid point is $N_{pav} + N_{r5d} - 2$ = 35,764. If we evaluate DOF in this manner, differences in Fig. 4a are found to be above 99% significant level almost all over the world. This way of evaluating statistical significance is apparently too lax due to the overestimation of DOF, because randomized time series with respect to each model might not be regarded as independent sample.

Therefore, we adopted more strict criteria for the estimation of DOF. Let the number of models which show the emergence of tipping year at a certain grid point for *pav* and that for *r5d* denoted by M_{pav} and M_{r5d}, respectively. At the grid point near Singapore, M_{pav} = 7 and M_{r5d} = 8. The difference between *r5d* and *pav* at this grid point is above 95% significant level which is lower than the previous estimation using N_{pav} and N_{r5d}. With this method, statistical significant areas are limited to equatorial and high-latitude regions (Fig. 4).

Text S3. Cumulative probability.

Cumulative probabilities integrated from 2010 to 2050 are calculated on a grid point base (Fig. S4) to estimate the possibility of emergence of tipping year. Figure S5 illustrates the differences of cumulative probabilities of *r5d* and *r1d* relative to *pav*. In case of RCP2.6 (Figs. S5a-b), cumulative probabilities of *r5d* and *r1d* are larger than those of *pav* in the subtropics and midlatitudes. These positive differences in probability are larger in higher emission scenarios (Figs. S5c-h) as compared with the lowest emission scenario (Fig. S5a-b). Especially, in the case of *r1d*

for RCP8.5 (Fig. S5h), the area of larger positive difference expands further to midlatitudes. This suggests that tipping year for intense precipitation is more likely to emerge than that for moderate and average precipitation in the subtropics and midlatitudes in case of the highest emission scenario. The dependency of difference of cumulative probability on precipitation indices and emission scenario (Fig. S5) is almost similar and consistent with that of tipping year (Fig. 4).

Text S4. Decomposition of simulated extreme precipitation change into thermodynamic and dynamic contribution.

To understand the physical mechanism of future extreme precipitation change, we apply a physical scaling diagnostic (Pfahl et al. 2017) which relates extreme precipitation amount to the vertical advection of moisture during an extreme event. The annual maximum of daily precipitation *r1d* at each grid point of climate model can be expressed as

$$r1d \simeq -\int \omega_a \frac{dq_s}{dp} dp \qquad (3)$$

where

ω_a ; vertical pressure velocity of ascending motion,

q_s ; saturation specific humidity

p ; pressure level.

A mass-weighted vertical integration is operated over all tropospheric levels only for ascending motion ($\omega_a < 0$). The troposphere is defined as all pressure levels below the highest level with a lapse rate smaller than 2 K km^{-1} and below 50 hPa. q_s is calculated from air temperature using a modified Tetens formula proposed by Simmons et al. (1999). Here we call the right-hand side of equation (3) as 'scaling diagnostic'. For the estimation of the scaling diagnostic, we use vertical profile of daily average vertical pressure velocity ω and air temperature on pressure levels at the location and on the day of occurrence of *r1d.* The scaling diagnostic can be interpreted as an estimate of the column integrated net condensation rate assuming a moist-adiabatic, saturated ascent of air parcels.

Figure S14a shows the distribution of *r1d* estimated from historical experiment for 20 years from 1981 to 2001 simulated by the MRI-CGCM3. Extreme precipitation events are concentrated over the tropics. Figure S14b shows the scaling diagnostic of *r1d* estimated from the same simulations as in Fig. S14a. The distribution of the scaling diagnostic (Fig. S14b, global average; 57.7 mm/day) is almost similar to that of *r1d* (Fig. S14a, global average; 56.7 mm/day). The spatial correlation between *r1d* (Fig. S14a) and the scaling diagnostic (Fig. S14b) is 0.986. The bias of global average of the scaling diagnostic is +1.0 mm/day which is +1.8 % relative to the global average of *r1d.*

Figure S14c depicts a mass-weighted vertically integrated q_s from the same simulations as in Fig. S14a on the day of occurrence of *r1d*. Column integrated moisture available for condensation over the tropics is much larger than over higher latitudes. This is consistent with large *r1d* (Fig. S14a) and corresponding large scaling diagnostic (Fig. S14b) over the tropics. Similarly, Fig. S14d shows a mass-weighted vertically integrated ω on the day of occurrence of *r1d.* Strong ascending motion (Fig. S14d) is much more concentrated over the tropics than over higher latitudes in contrast to saturated specific humidity (Fig. S14c) where the distribution is nearly homogeneous in longitudinal direction. The locations of strong ascending motion are well matched with those of large *r1d* (Fig. S14a). This suggest that extreme precipitation events over the tropics can be attributed to strong ascending motion rather than abundant moisture.

Figure S14e illustrates future *r1d* from RCP8.5 experiment for 20 years from 2081 to 2100 simulated by the MRI-CGCM3. The distribution of scaling diagnostic for future *r1d* (Fig. S14f) is almost similar to that of *r1d* (Fig. S14e) although the global average estimated by scaling diagnostic overestimates *r1d*.

Figure S14g shows the scaling diagnostic using future vertical profile of q_s but with present-day vertical profile of ω on the day of occurrence of *r1d*. This can be regarded as thermodynamical effect or contribution for future *r1d*. Conversely, Fig. S14h is the scaling diagnostic using present-day vertical profile of q_s but with future vertical profile of ω on the day of occurrence of *r1d*. This can be regarded as dynamical effect or contribution. The spatial pattern of thermodynamical effect (Fig. S14g) approximately resembles to that of the total future scaling diagnostic (Fig. S14f). The global average of scaling diagnostic for thermodynamical contribution is 67.8 mm day^{-1} (Fig. S14g) which is 84.8 % of the global average of total scaling diagnostic 80.0 mm day^{-1} (Fig. S14f). Similarly, the global average of scaling diagnostic for dynamical contribution is 64.9 mm day^{-1} (Fig. S14h) which is 81.1 % of the global average of total scaling diagnostic 80.0 mm day^{-1} (Fig. S14f). Therefore, the relative magnitude of thermodynamical and dynamical contributions is nearly same in terms of global average. Since the scaling diagnostic is a non-linear function of q_s and ω, the sum of thermodynamical (Fig. S14g) and dynamical (Fig. S14h) contributions does not necessarily match with the total scaling diagnostic (Fig. S14f).

Figure S14i depicts the future distribution of the vertically integrated saturated q_s from the same simulations as in Fig. S14e on the day of occurrence of *r1d*. The global average of vertically integrated q_s in the future simulations (Fig.S14i, 5.60 g Kg^{-1}) is larger than that of the present-day simulations (Fig.S14c, 4.51 g Kg^{-1}). Figure S14j shows the future distribution of the vertically integrated ω from the same simulations as in Fig. S14i. The global average of larger negative ω in the future simulation (Fig.S14j, -41.2 10^{-2}Pa s^{-1}) as compared with that of the present-day simulation (Fig.S14d, -35.7 10^{-2} Pa s^{-1}) suggests the increase of strong ascending motion.

Figure S14h shows the ratio of the future change in *r1d* (Fig.S14e minus Fig.S14a) to the present-day climatology (Fig.S14a) at each grid points. *r1d* increases almost all regions in the globe. Increase of *r1d* in the tropics is larger than in higher latitudes. Similar tendency is found in terms of the scaling diagnostic (Fig. S14m). Thermodynamical contribution (Fig. S14n) to the change in total scaling diagnostic (Fig. S14m) is estimated as the difference between future thermodynamical contribution (Fig. S14g) and present-day scaling diagnostic (Fig. S14b) normalized by present-day scaling diagnostic (Fig. S14b) at each grid point. Thermodynamical contribution to future change in total scaling diagnostic is almost positive in the globe, but the future positive change due to thermodynamical effect is smaller in the tropics than higher latitudes (Fig. S14n) in contrast to to the tendency in the total scaling diagnostic (Fig. S14m). On the contrary, dynamical contribution is positive over the tropic while it is negative in higher latitudes (Fig. S14o). Larger increase of total scaling diagnostic (Fig. S14m) over the tropics can be attributed to the larger dynamical contribution over the tropics (Fig. S14n). On the other hand, increase of total scaling diagnostic (Fig. S14m) over higher latitudes can be attributed to the large positive change in thermodynamical contribution (Fig. S14n) and small negative change in dynamical contribution (Fig. S14o) over higher latitudes.

The global increase of thermodynamical contribution (Fig. S14n) is consistent with global increase of moisture (Fig. S14p) except for the Antarctica. The large increase of dynamical contribution over the tropics (Fig. S14o) originates from large increase of ascending motion over the tropics (Fig. S14q).

Figure S15 illustrates the relationship between the scaling diagnostic and tipping year of *r1d* for RCP8.5 based on 8 CMIP5 models in Table S5. Fig S15a indicates that models with stronger thermodynamical effect tend to project an earlier tipping year. On the contrary, projected tipping year is not sensitive to dynamical effect (Fig. S15b). Projected tipping year also depends on specific humidity (Fig. S15c) which is consistent with dependency on thermodynamical effect (Fig. S15a). On the other hand, projected tipping year is not sensitive to vertical velocity (Fig. S15d) which is consistent with Fig. S15b. Judging from the analyses of scaling diagnostic, tipping year of intense precipitation is affected by change in moisture rather than largescale circulation.

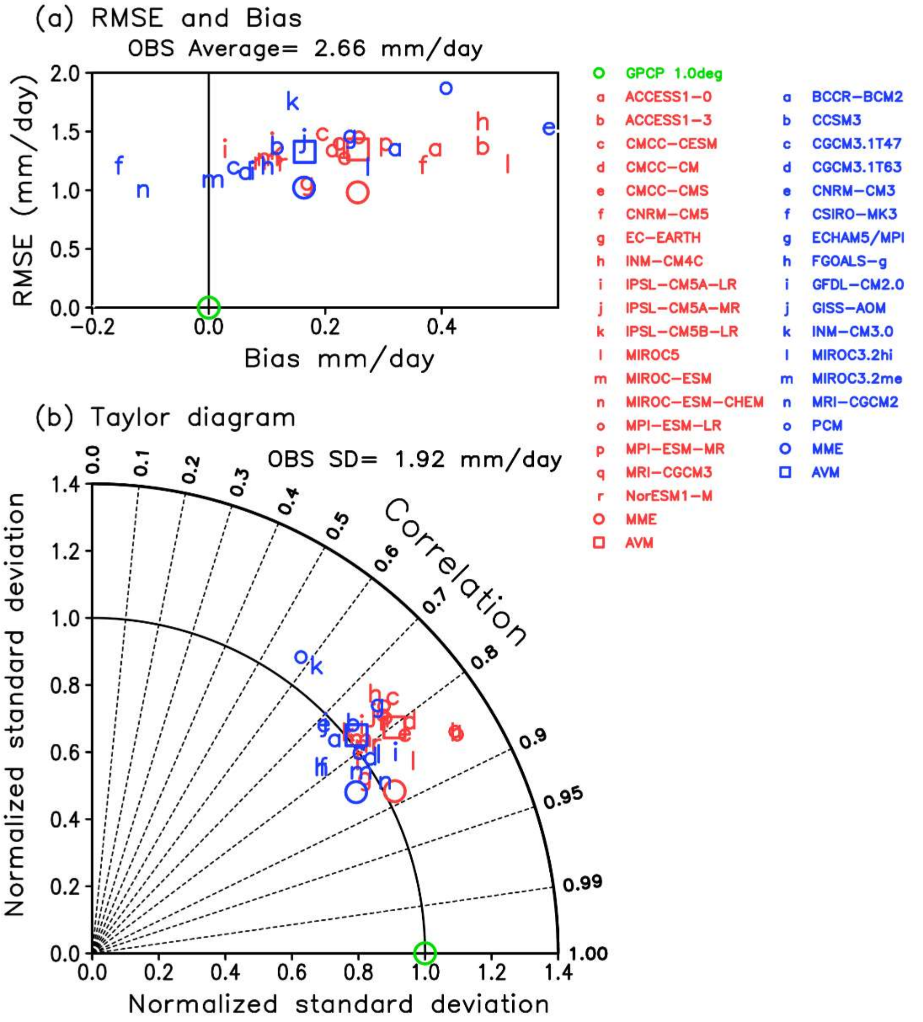

Figure S1. Reproducibility of the global distribution of annual mean precipitation *pav* by the CMIP5 and CMIP3 models. The green circles indicate the observation of Global Precipitation Climatology Project (GPCP) 1-Degree Daily (1DD) Version 1.2 data (1979-2013, 17 years; Table S4; Huffman et al. 2001). The red characters and marks denote the CMIP5 models used in this study (Table S1 and S2). The blue characters and marks denote the CMIP3 models (Table S3). The circle marks indicate the skill of precipitation distribution obtained from the Multi-Model Ensemble (MME) average. The square marks indicate the average skill of the individual models (AVM). The 20-year periods from 1981 to 2000 of historical experiments were used for model verification. (**a**) The Root Mean Square Error (RMSE) and bias (mm day^{-1}). The global average of observation is shown above the panel. (**b**) The Taylor diagram for displaying pattern statistics (Taylor 2001). The radial distance from the origin is proportional to the standard deviation of a simulated pattern normalized by the observed standard deviation. The spatial correlation coefficient between the observed and simulated fields is given by angle from y-axis. The standard deviation of the observation in the global domain is shown above the panel. Model data were interpolated to the location of grid points (1 degree in longitude and latitude) of GPCP 1DD data.

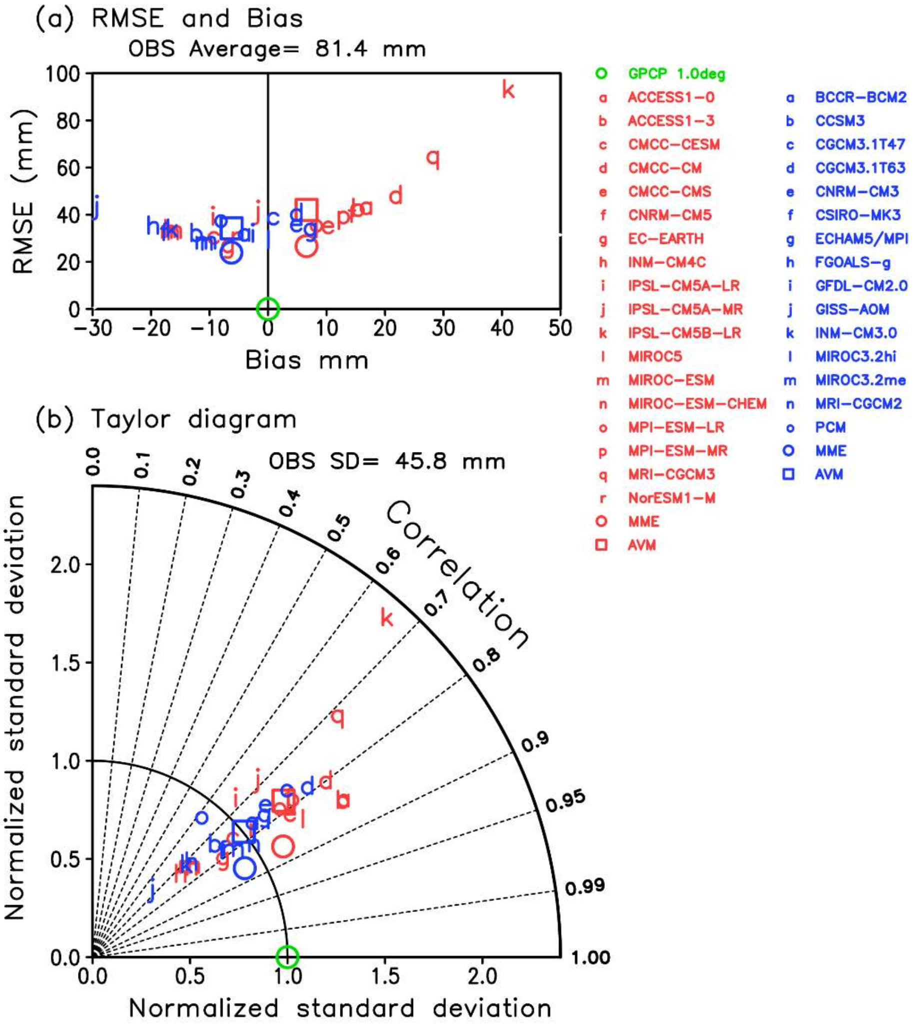

Figure S2. Reproducibility of the global distribution of the annual maximum of daily precipitation *r5d* by the CMIP5 and CMIP3 models. Figure format is the same as Fig. S1.

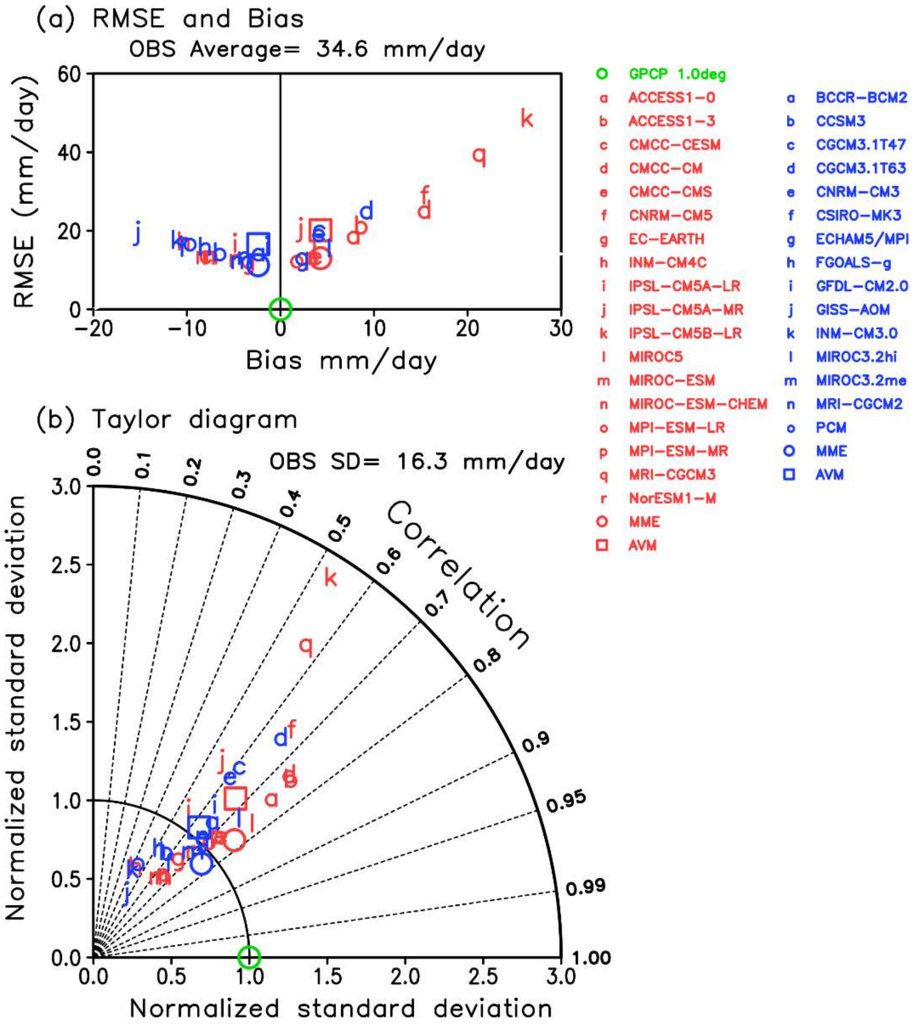

Figure S3. Reproducibility of the global distribution of the annual maximum of daily precipitation *r1d* by the CMIP5 and CMIP3 models. Figure format is the same as Fig. S1.

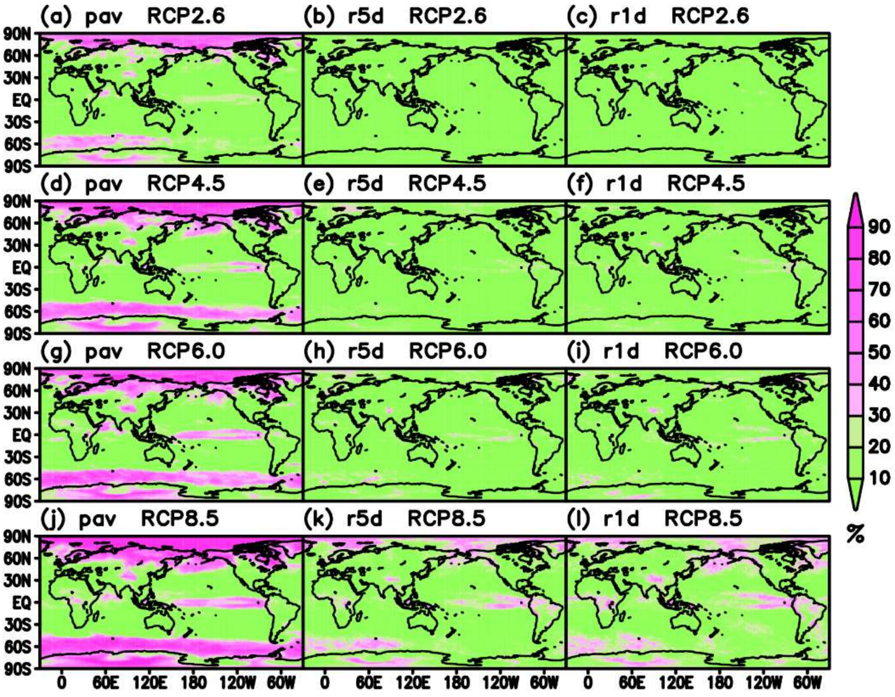

Figure S4. Cumulative probability of tipping year before 2050. Cumulative probabilities (%) of PDF for MME average from 2010 to 2050 are calculated with respect to three precipitation indices and for four emission scenarios.

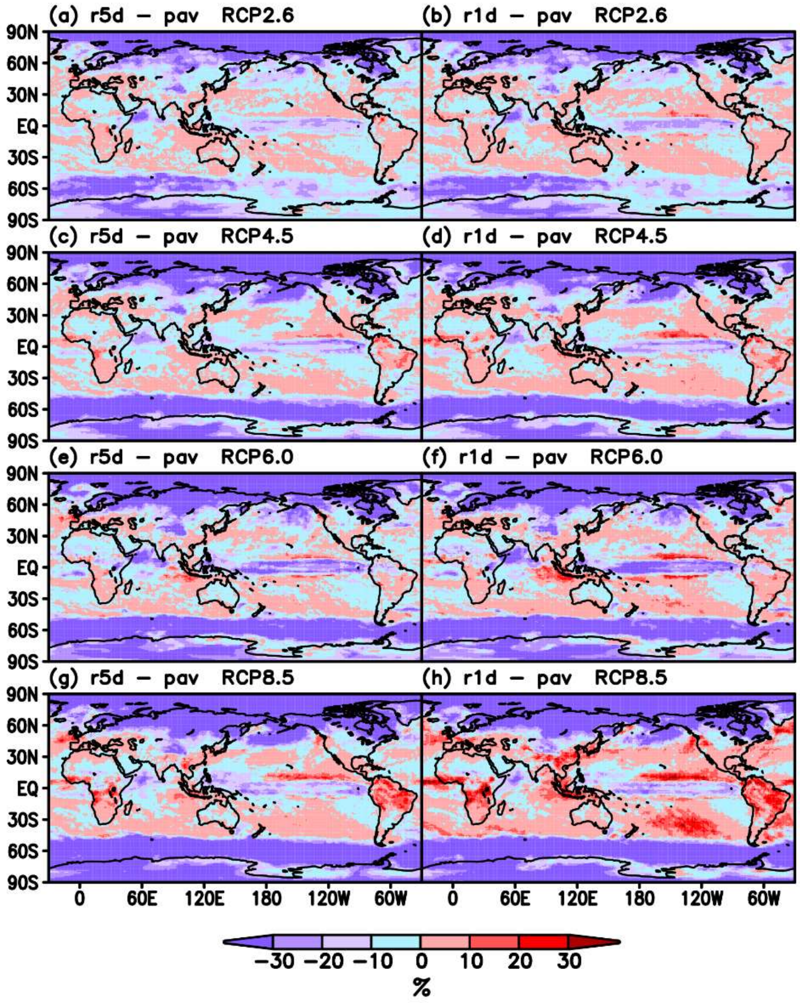

Figure S5. Differences of cumulative probability of tipping year before 2050. Cumulative probabilities (%) from 2010 to 2050 are calculated at each grid point and the difference of *r5d* - *pav* and *r1d* - *pav* are shown.

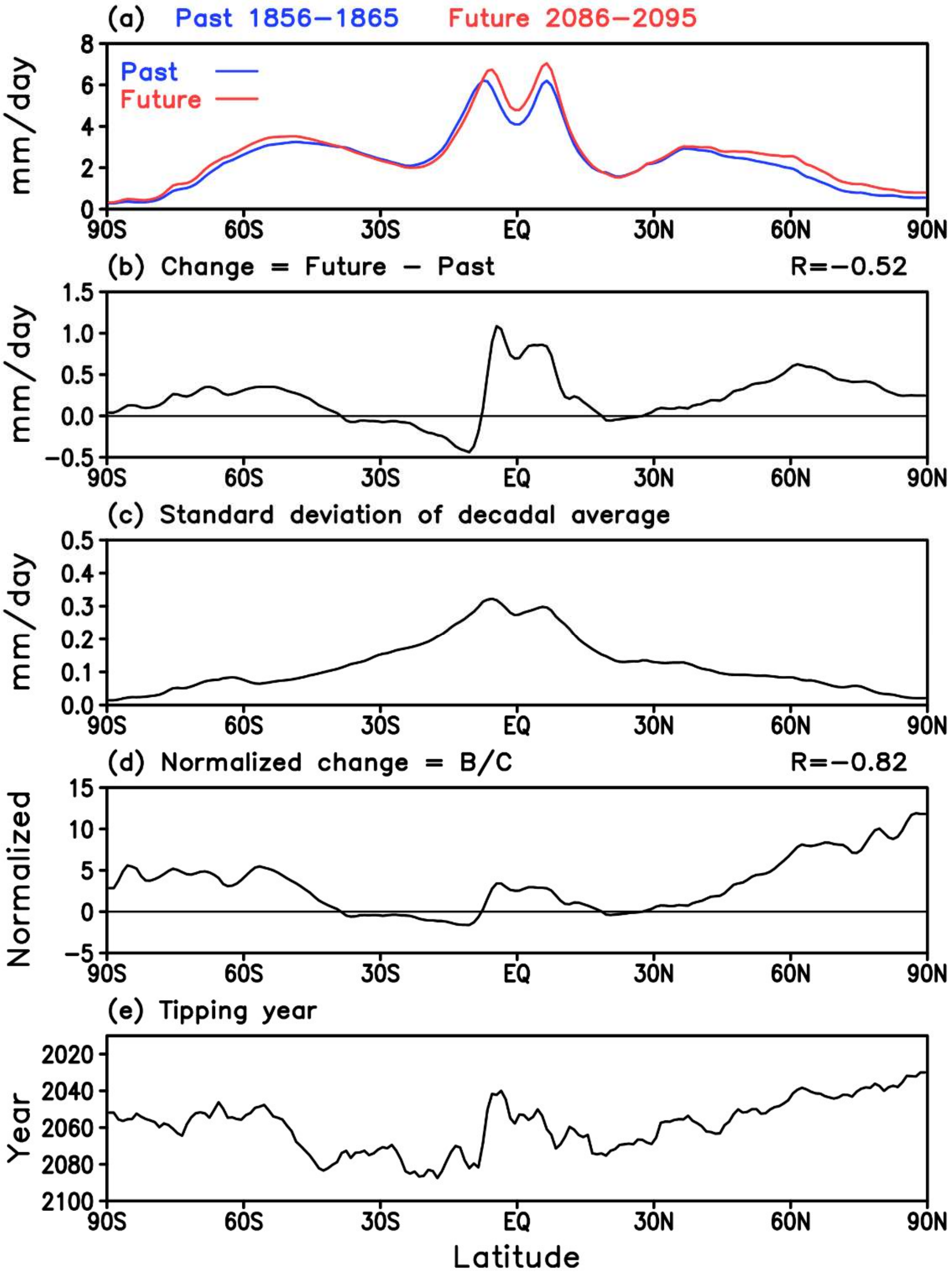

Figure S6. Zonally averaged precipitation change and the tipping year for *pav*. Simulations by the MRI-CGCM3 for RCP8,5 is used. (**a**) Precipitation of past for 1856-1865 (blue) and future for 2086-2095 (red). (**b**) Change = Future - Past. Value of R is the spatial correlation coefficient between change and tipping year (E). (**c**) Decadal natural variability S_d . (**d**) Precipitation change normalized by S_d as **b** /**c**. Value of R is the spatial correlation coefficient between normalized change and the tipping year **c**. (**e**) The tipping year. Note that vertical axis is reversed.

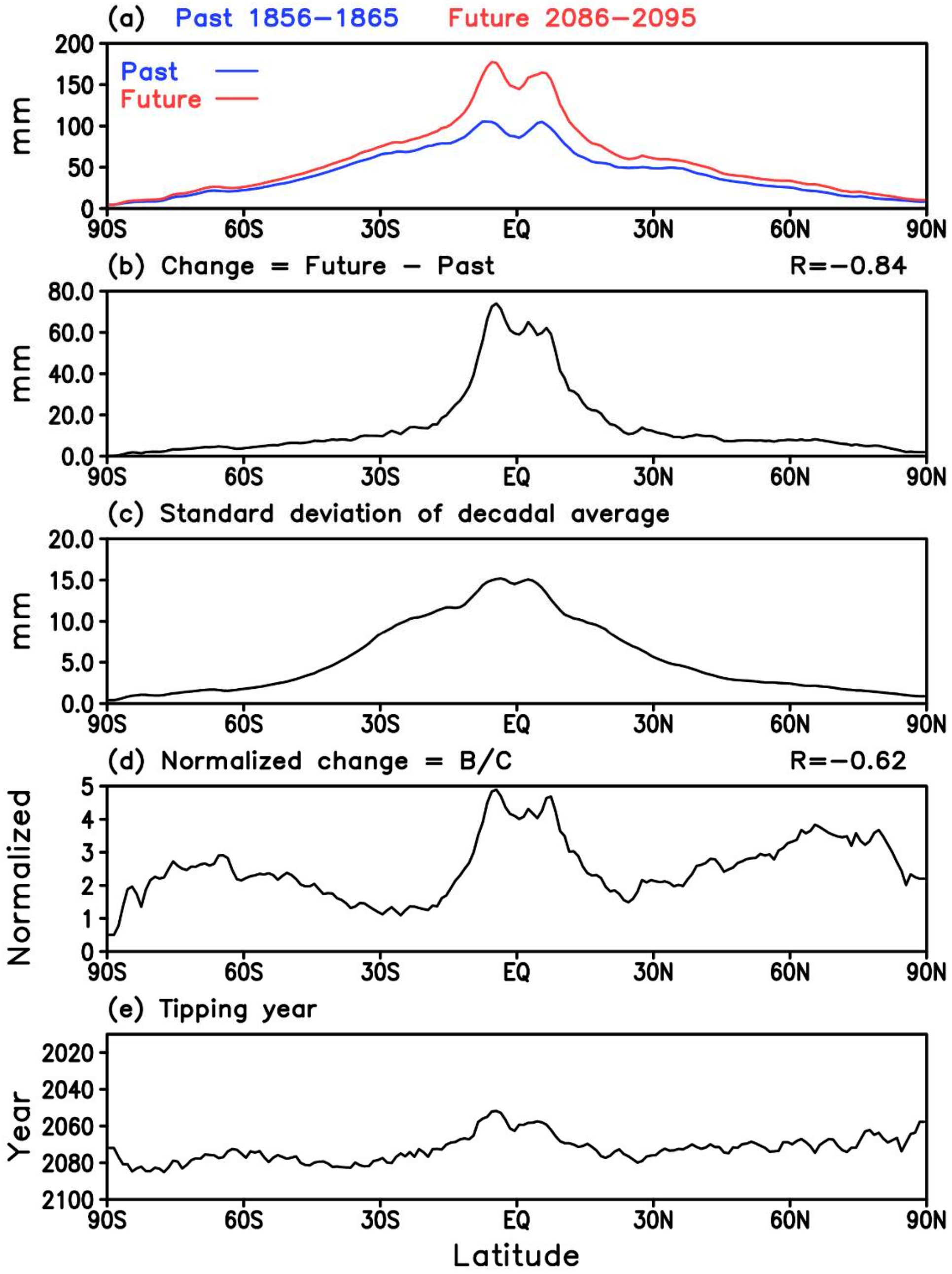

Figure S7. Zonally averaged precipitation change and the tipping year for *r1d*. Simulations by the MRI-CGCM3 for RCP8,5 is used. Figure format is the same as Fig. S6.

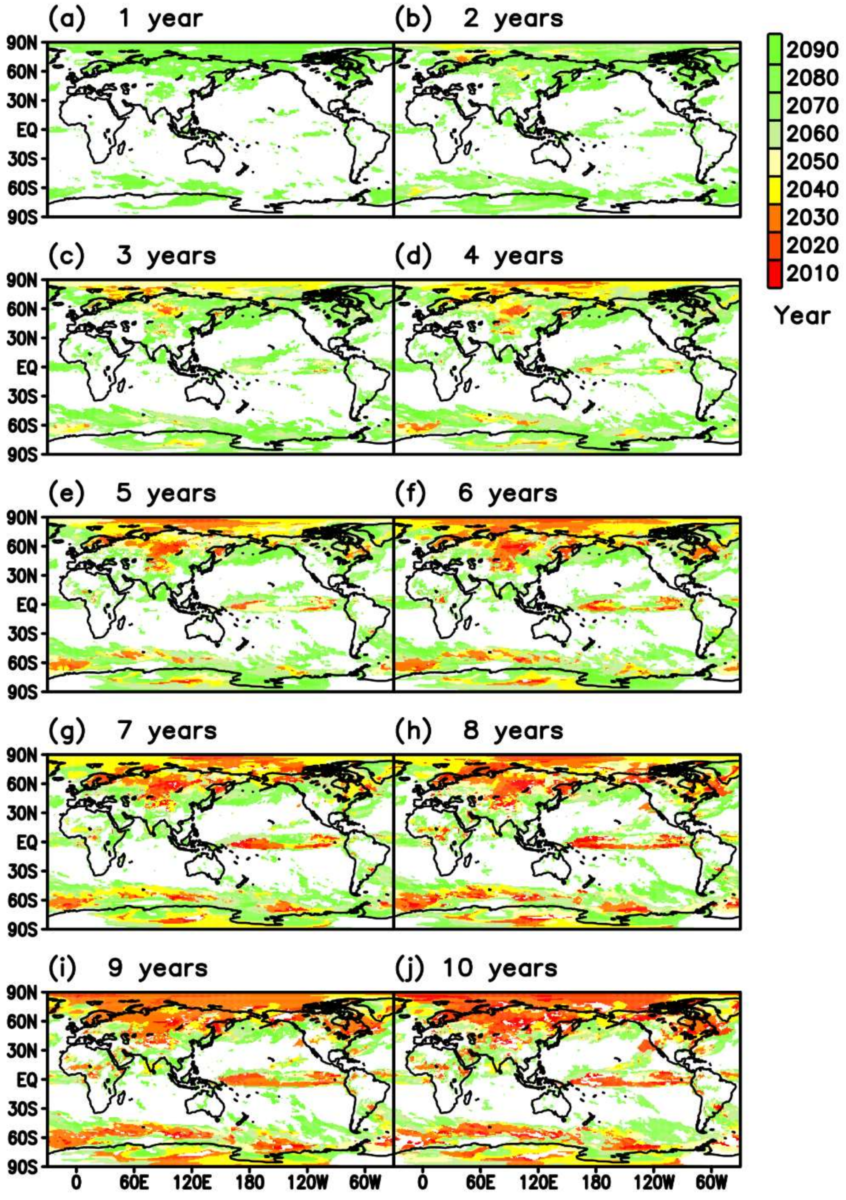

Figure S8. Dependence of the tipping year on time scale. Simulations by the MRI-CGCM3 for RCP8.5 is used. (**a**) The tipping year of *pav* using year-to-year time series. (**b**) Same as **a**, but for two-year average of annual precipitation. (**c**-**j**) Same as **b**, but for different averaging periods.

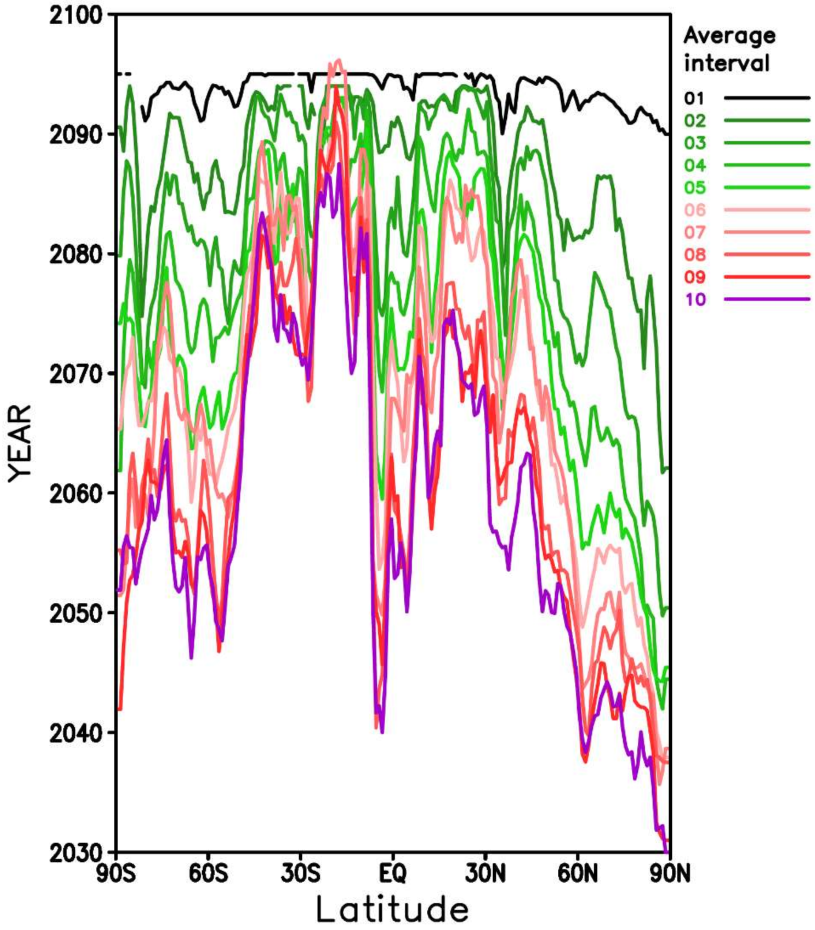

Figure S9. Dependence of the zonal averaged tipping year on time scale. Simulations by the MRI-CGCM3 for RCP8.5 is used. Zonal average of Fig. S8 is shown.

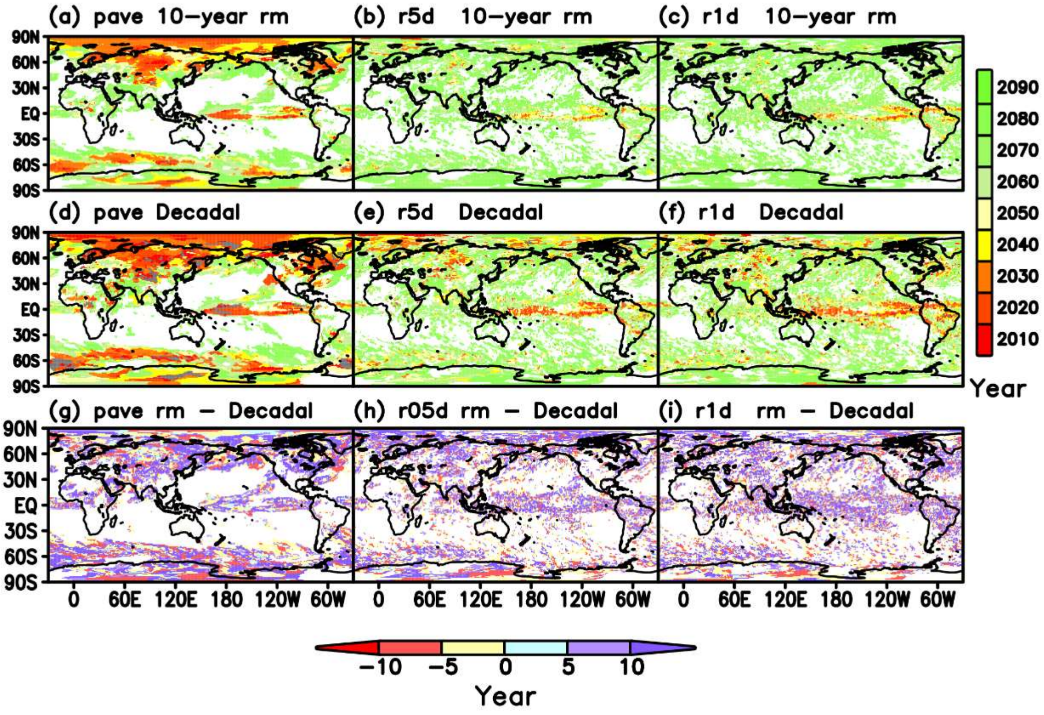

Figure S10. Comparison between tipping year based on 10-year running mean and decadal mean. Simulations by the MRI-CGCM3 for RCP8.5 is used. The white regions indicate a missing of tipping year. (**a**) *pav* based on 10-year running mean. (**b**) Same as **a** but for *r5d*. (**c**) Same as **a** but for *r1d*. (**d**-**e**) Same as **a**-**c** but for decadal average. (**g**) Difference between tipping year based on 10-year running mean and that on decadal average for *pave*. (**h**) Same as **g** but for *r5d*. (**i**) Same as **g** but for *r5d*.

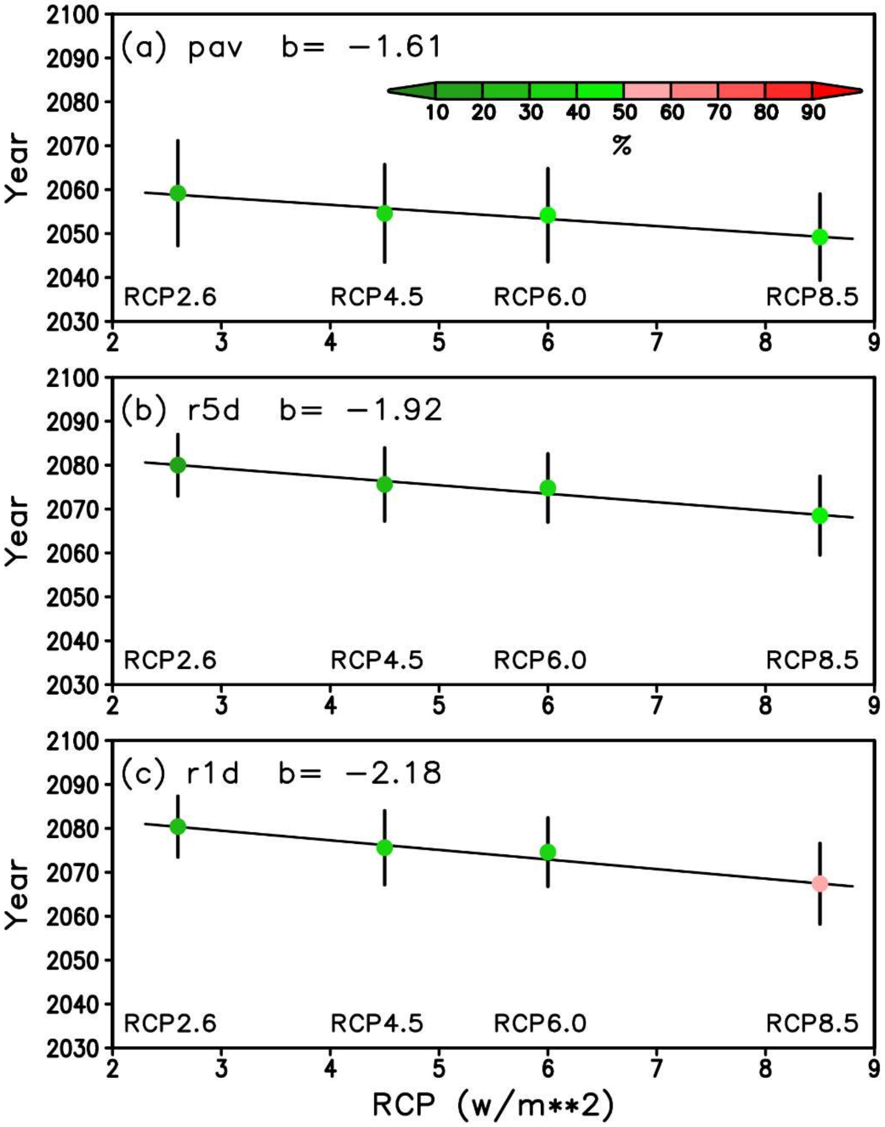

Figure S11. Dependence of tipping year on RCP scenario. (**a**) *pav*. Vertical position of closed circle shows the expectation value of the PDF of the MME average TY_{av}. The vertical bar shows the range of one standard deviation of the PDF TY_{sd}. Color shows the existence rate of the tipping year E (%). The slant line shows the linear regressed line using E as weighting factor. The value of 'b' is inclination. Unit is year (w $m^{-2})^{-1}$. (**b**) *r5d*. (**c**) *r1d*.

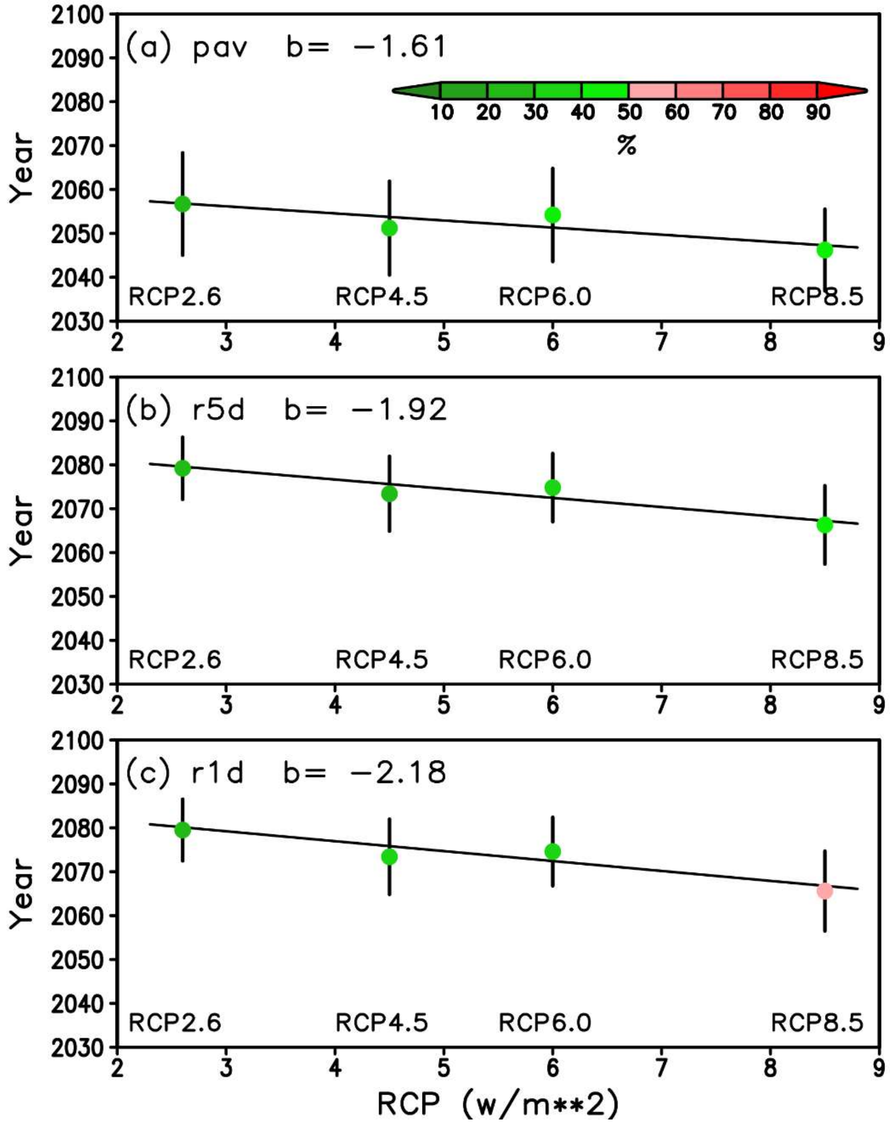

Figure S12. Dependence of tipping year on RCP scenario using 7 Models. Same as Fig. S11 but for limited 7 CMIP5 models (Table S1) which save all the four RCP data.

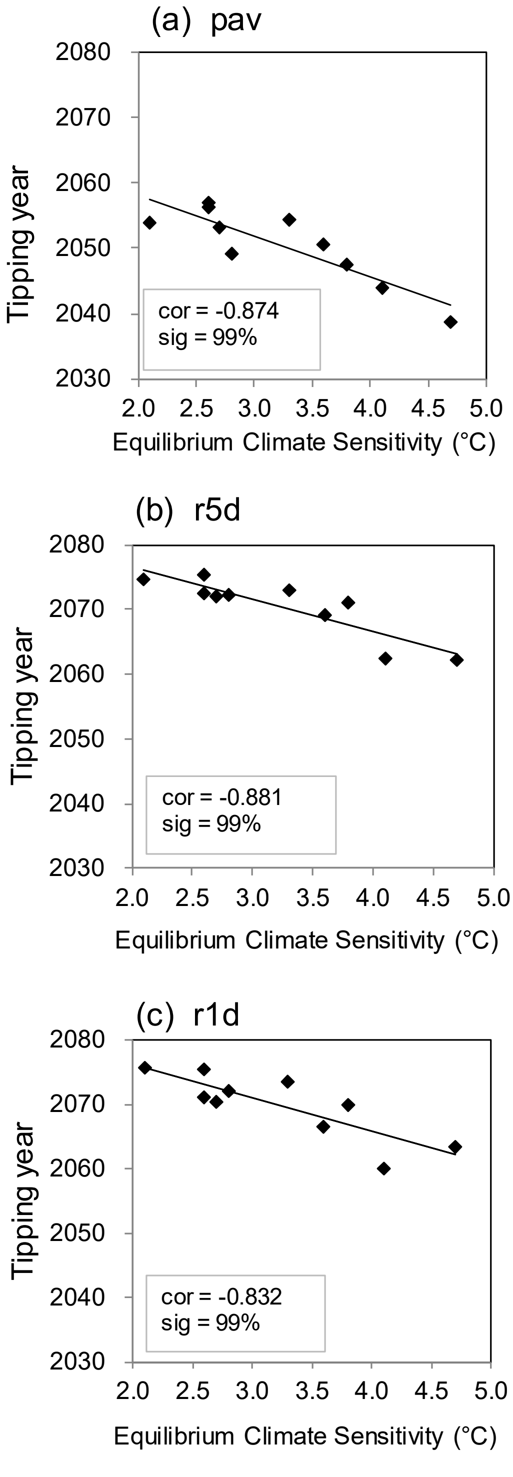

Figure S13. Relation between the equilibrium climate sensitivity (°C) and the global averaged tipping year (year) for RCP8.5. Out of the 18 models used in this study, the climate sensitivities of 10 models (Table S5) are only available from the Table 9.5 of Flato et al. (2013). The slant black line is linear regression fit. Value of 'cor' is correlation coefficient. Value of 'sig' is the statistical significance level of correlation. (**a**) *pav*. (**b**) *r5d*. (**c**) *r1d*.

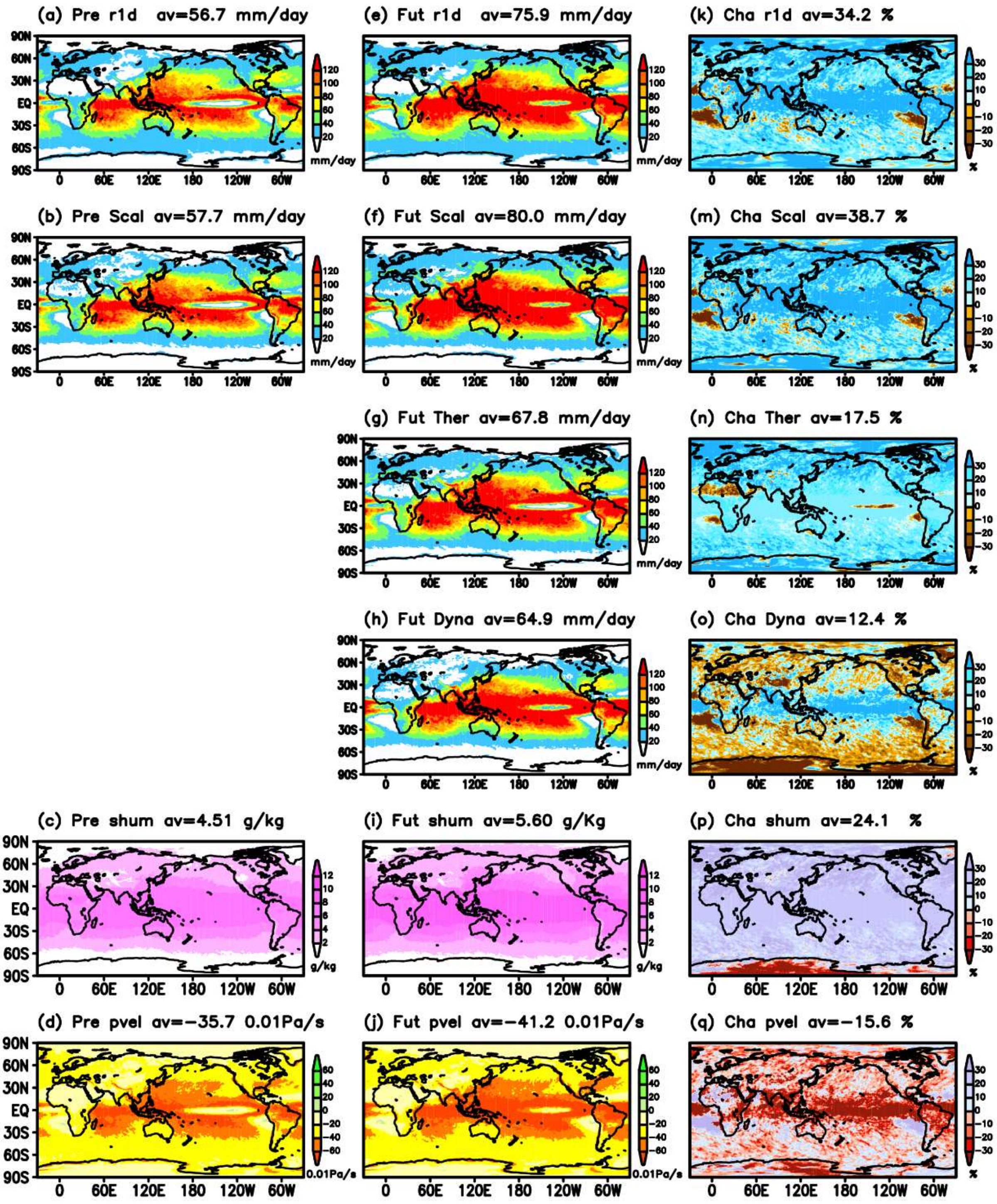
(a) Pre r1d av=56.7 mm/day
(e) Fut r1d av=75.9 mm/day
(k) Cha r1d av=34.2 %
(b) Pre Scal av=57.7 mm/day
(f) Fut Scal av=80.0 mm/day
(m) Cha Scal av=38.7 %
(g) Fut Ther av=67.8 mm/day
(n) Cha Ther av=17.5 %
(h) Fut Dyna av=64.9 mm/day
(o) Cha Dyna av=12.4 %
(c) Pre shum av=4.51 g/kg
(i) Fut shum av=5.60 g/Kg
(p) Cha shum av=24.1 %
(d) Pre pvel av=−35.7 0.01Pa/s
(j) Fut pvel av=−41.2 0.01Pa/s
(q) Cha pvel av=−15.6 %
mm/day
g/kg
0.01Pa/s
90N
60N
30N
EQ
30S
60S
90S
0
60E
120E
180
120W
60W

Figure S14. Decomposition of the annual maximum of daily precipitation r1d change into thermodynamic and dynamic contribution. The historical experiment of 20 years from 1981 to 2000 (the present-day simulation) and RCP8.5 experiment of 20 years from 2081 to 2100 (the future simulation) by the MRI-CGCM3 were used for analyses. Value of 'av' at the top of each panels denotes the global average. (**a**) *r1d* for the present-day simulation. Unit is mm day^{-1}. (**b**) The scaling diagnostic calculated by the equation (3) for the present-day simulation. Unit is mm day^{-1}. (**c**) The vertically integrated saturation specific humidity q_s on the day of occurrence of *r1d* for the present-day simulation. Unit is g Kg^{-1}. (**d**) Same as **c** but for vertical pressure velocity ω. Unit is 10^{-2} $Pa\ s^{-1}$. (**e**) Same as **a** but for the future simulation. (**f**) Same as **a** but for the future simulation. (**g**) Thermodynamical contribution to the future scaling diagnostic. (**h**) Same as **g** but for dynamical contribution. (**i**) Same as **c** but for the future simulation. (**j**) Same as **d** but for the future simulation. (**k**) Future change normalized by the present-day climatology at each grid point. (**e** minus **a**)/**a**. Unit is %. (**m**) Same as k but for the scaling diagnostic. (**n**) Future change of thermodynamical contribution. (**g** minus **b**)/**b**. (**o**) Same as **n** but for dynamical contribution. (**p**) Same as **k** but for q_s. (**q**) Same as **p** but for ω.

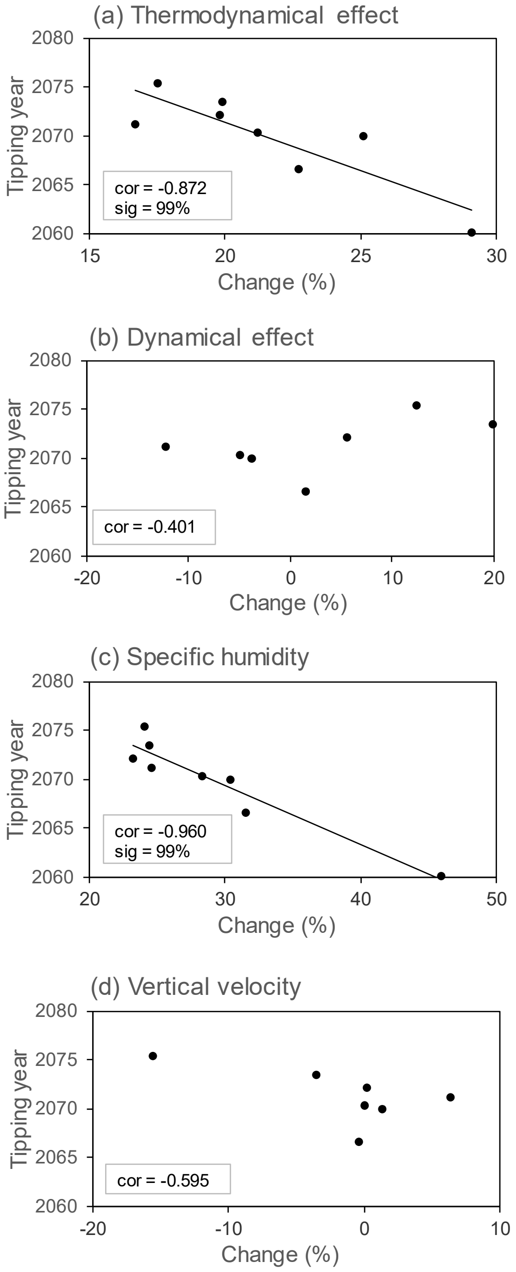

Figure S15. Relationship between the scaling diagnostic and tipping year of *r1d* for RCP8.5. Eight CMIP5 models in Table S5 are used. Daily 3-dimensional data of ω which is required for the calculation of the scaling diagnostic defined by the equation (1) were not available for INM-CM4 and MIROC-ESM. (**a**) Dependence of global averaged tipping years on the future changes in the global averaged thermodynamical contribution (17.5 % in the case of MRI-CGCM3, Fig. S14n). The slant line is linear regression fit. Value of 'cor' is correlation coefficient. Value of 'sig' is the statistical significance level of correlation coefficient. (**b**) Same as **a** but for dynamical contribution (12.4 % in the case of MRI-CGCM3, Fig. S14o). The correlation coefficient is not statistically significant. (**c**) Same as **a** but for global average of vertically integrated q_s on the day of occurrence of *r1d*. (**d**) Same as **c** but for ω.

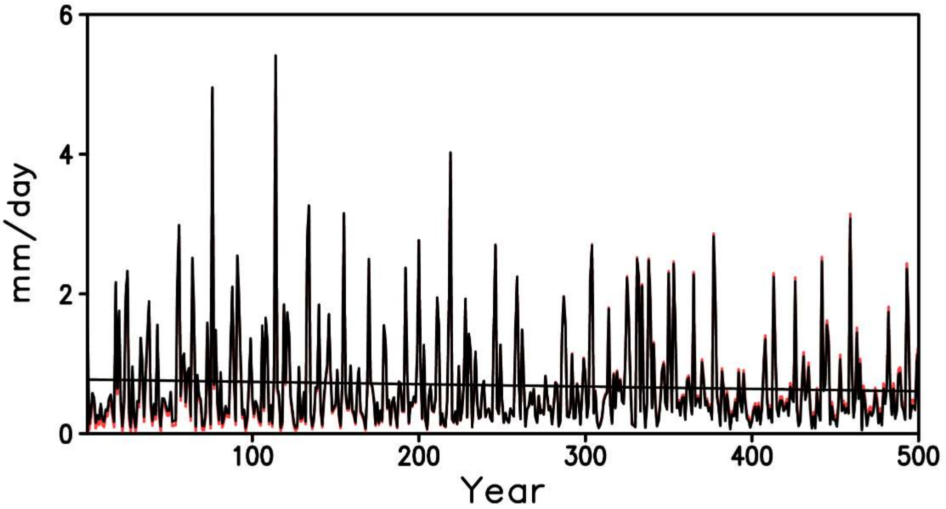

Figure S16. An example of time series from the piControl experiment. The black line is *pav* simulated by the MRI-CGCM3 at the grid point (179.5°W, 0.5°N; X mark in Fig. 1a, S8). The black slant straight line shows the linear rend (-0.03 mm day^{-1} $century^{-1}$) which is not statistically significant above 99 % level. The red line is detrended time series which almost overlaps with the original time series (black).

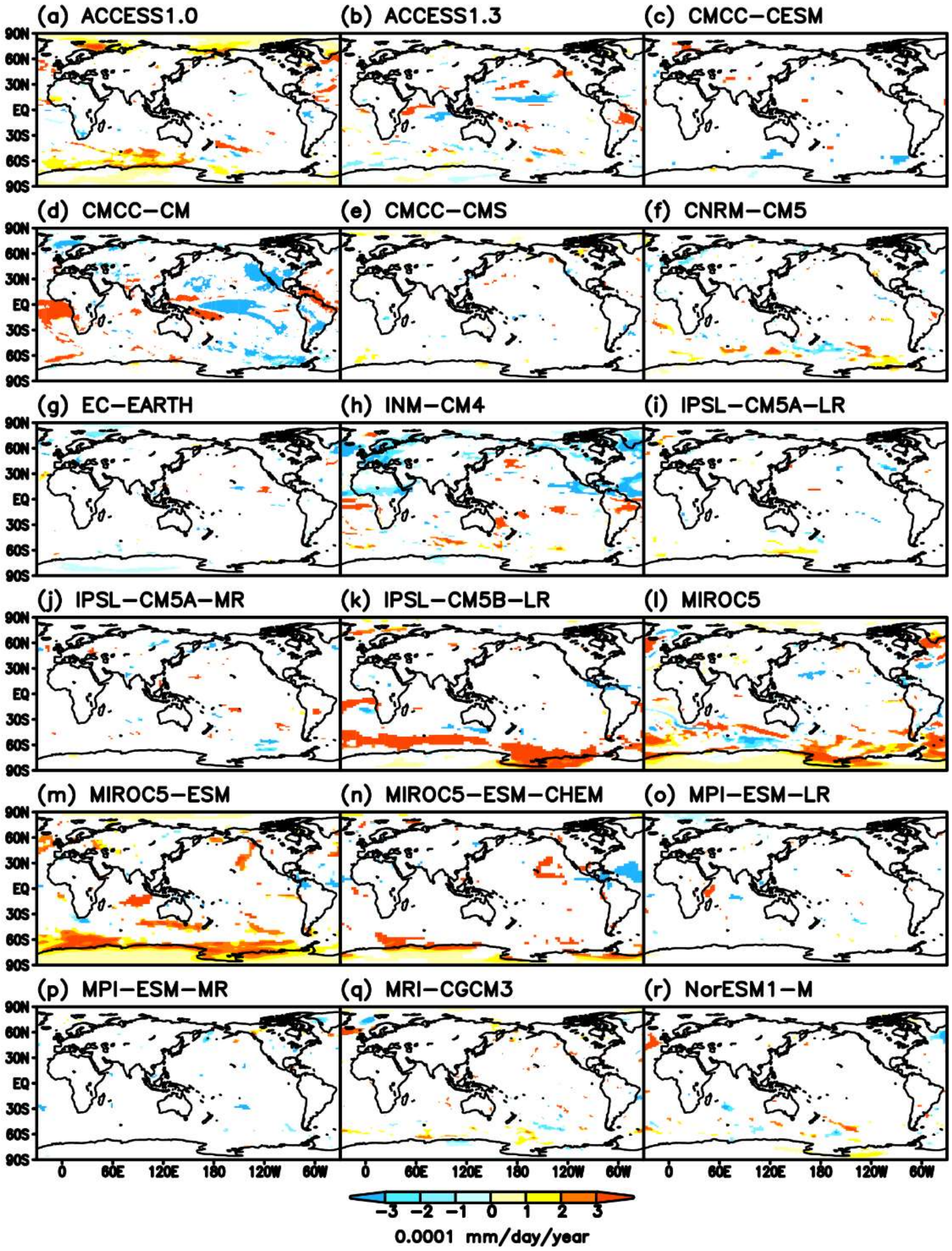

Figure S17. Trend of *pav* simulated by the piControl experiments of the CMIP5 models. Regions with a significance level exceeding 95% are only colored. Unit is 10^{-4} mm day^{-1} year^{-1}. The lengths of simulated period are shown in Table S1.

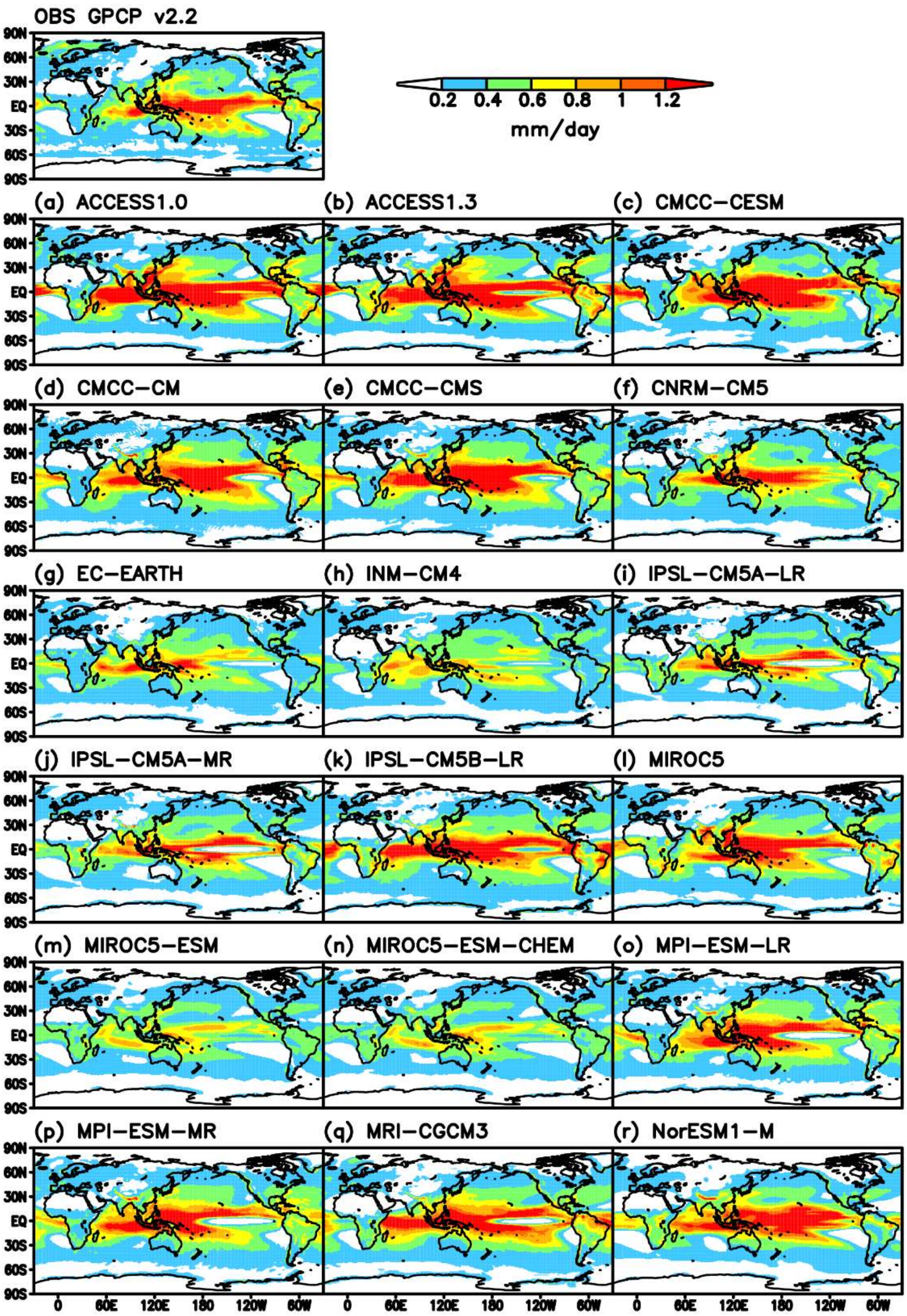

Figure S18. Year-to-year natural variability S_y for *pav*. The top panel shows the observation of GPCP Version 2.2 data (1979-2010, 32 years; Table S4; Adler et al. 20103). We created an artificial 200-year long data by random sampling. We divided the whole 200-year period into 20 ten-year periods and calculated standard deviations of year-to-year variabilities for each period. Then we averaged 20 standard deviations. (**a**-**r**) Simulated variability by the piControl experiments of the CMIP5 models.

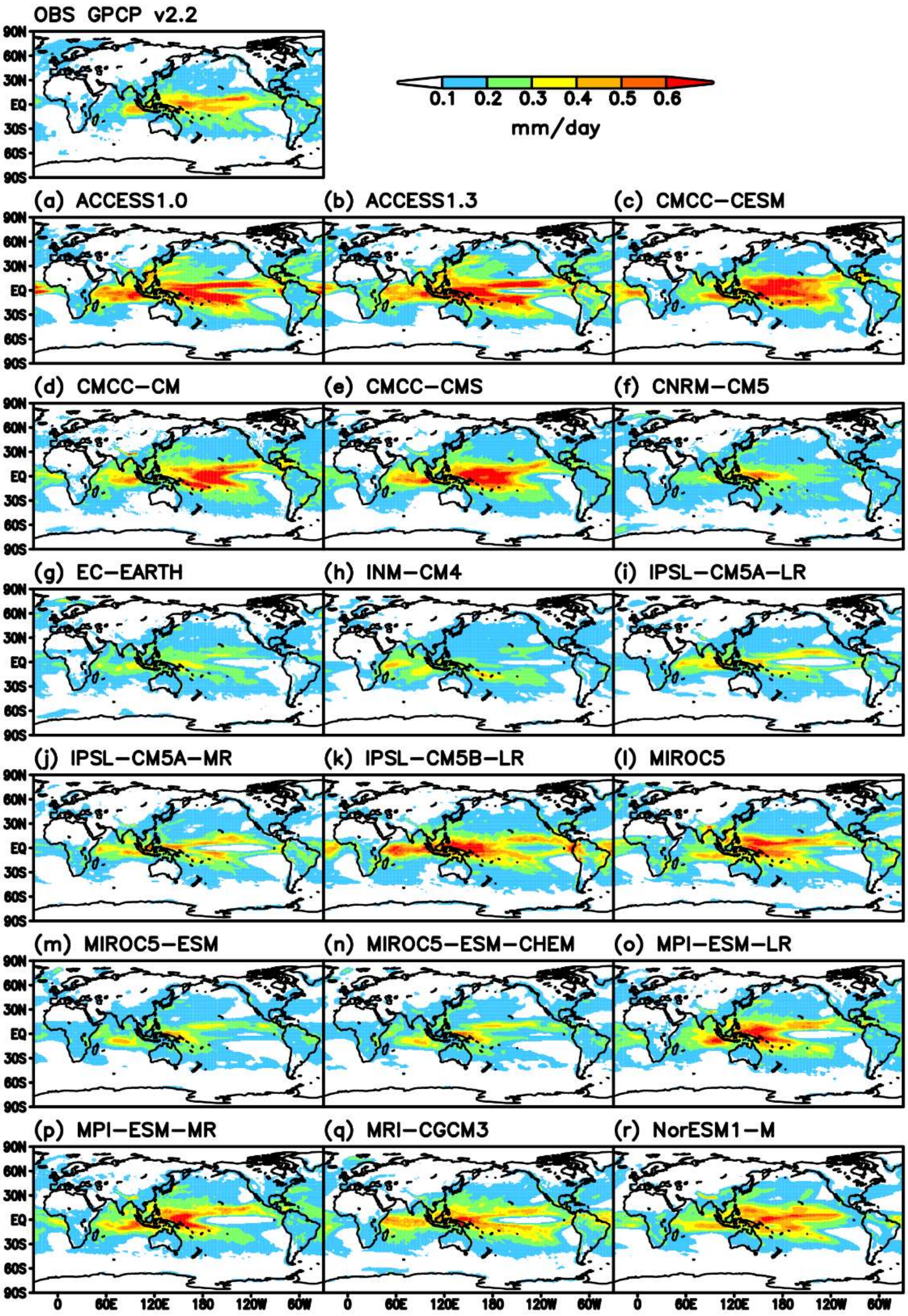

Figure S19. Decadal natural variability S_d of *pav*. The top panel shows the observation of GPCP Version 2.2 data. As in Fig. S6, artificial 200-year long data is used. Standard deviations are calculated from 20 ten-year averages. (**a**-**r**) Simulated variability by the piControl experiments of the CMP5 models.

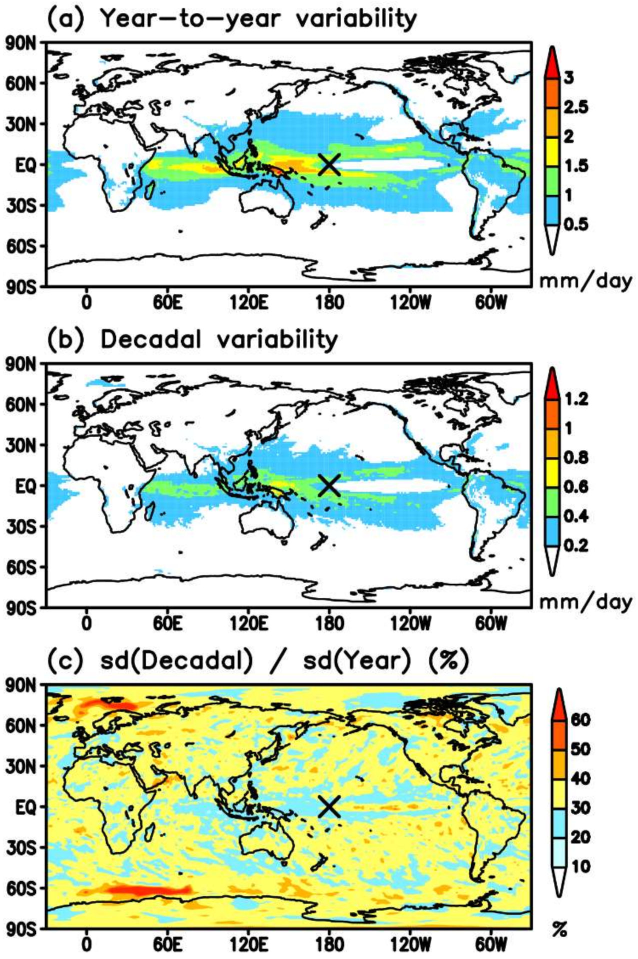

Figure S20. Distribution of the natural variability of *pav*. The 500-year piControl experiments from the MRI-CGCM3 were used here. The mark × indicates the grid point used in Fig. 1 and S4. (**a**) Standard deviation of year-to-year variability S_y. (**b**) Standard deviation of decadal variability S_d. (**c**) Ratio (%) of decadal variability **b** to year-to-year variability **a** as S_d / S_y .

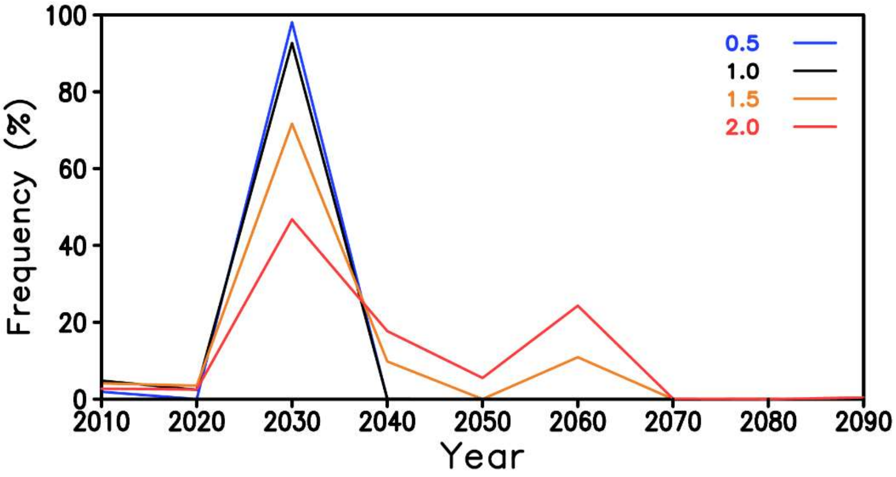

Figure S21. Dependence of the tipping year on the magnitude of decadal natural variability $\boldsymbol{V_d}$**.** The PDFs for *pav* were calculated using the 10,000 randomized time series at the grid point (179.5°W, 0.5°N marked with × in the inset map of Fig. 1a) from the historical and RCP8.5 experiments by the MRI-CGCM3, changing the magnitude of V_d as 0.5 V_d (blue), 1.0 V_d (black, default), 1.5 V_d (orange) and 2.0 V_d (red).

Table S1 Number of years simulated by 18 CMIP5[a] models used in this study.

No	Label used in Figures in Supplementary Materials	Model name[b]	Preindustrial controle experiment 'piControl'	Historical experiment 'historical' 1856-2005	Future Representative Concentration Pathways (RCP) experiment 2006-2095			
					RCP2.6	RCP4.5	RCP6.0	RCP8.5
1	a	ACCESS1.0	500	150	-[c]	90	-	90
2	b	ACCESS1.3	500	150	-	90	-	90
3	c	CMCC-CESM	270	150	-	-	-	90
4	d	CMCC-CM	330	150	-	90	-	90
5	e	CMCC-CMS	500	150	-	90	-	90
6	f	CNRM-CM5	500	150	90	90	-	90
7	g	EC-EARTH	450	150	-	-	-	90
8	h	INM-CM4	500	150	-	90	-	90
9	i	IPSL-CM5A-LR*	500	150	90	90	90	90
10	j	IPSL-CM5A-MR*	300	150	90	90	90	90
11	k	IPSL-CM5B-LR	300	150	-	90	-	90
12	l	MIROC5*	500	150	90	90	90	90
13	m	MIROC-ESM*	500	150	90	90	90	90
14	n	MIROC-ESM-CHEM*	250	150	90	90	90	90
15	o	MPI-ESM-LR	500	150	90	90	-	90
16	p	MPI-ESM-MR	500	150	90	90	-	90
17	q	MRI-CGCM3*	500	150	90	90	90	90
18	r	NorESM1-M*	500	150	90	90	90	90
		Total years[d]	7900	2700	900	1440	630	1620
		Number of models	18	18	10	16	7	18

[a] CMIP5: the fifith phase of the Coupled Model Intercmparison Project

[b] Name in Table 9.A.1 in Flato et al. (2013)

[c] Data are not available.

[d] Grand total: 15,190 years

* Models saving all the four RCP data ; 7 models

Table S2 Features of 18 CMIP5[a] models used

No	Label used in Extended Data Figures	Model name	Atmosphere			Ocean		
			Number of grids in longitude	Number of grids in latitude	Number of vertical levels	Number of grids in longitude	Number of grids in latitude	Number of vertical levels
1	a	ACCESS1.0	192	145	38	360	180	50
2	b	ACCESS1.3	192	145	38	360	180	50
3	c	CMCC-CESM	96	48	39	180	90	31
4	d	CMCC-CM	480	240	31	180	90	31
5	e	CMCC-CMS	192	96	95	180	90	31
6	f	CNRM-CM5	256	128	31	514	257	42
7	g	EC-EARTH	320	160	62	360	180	31
8	h	INM-CM4	180	120	21	360	360	40
9	i	IPSL-CM5A-LR	96	95	39	180	90	31
10	j	IPSL-CM5A-MR	144	143	39	180	90	31
11	k	IPSL-CM5B-LR	96	95	39	180	90	31
12	l	MIROC5	256	128	40	257	129	50
13	m	MIROC-ESM	128	64	80	257	129	44
14	n	MIROC-ESM-CHEM	128	64	80	257	129	44
15	o	MPI-ESM-LR	192	96	47	240	120	40
16	p	MPI-ESM-MR	192	96	95	900	450	40
17	q	MRI-CGCM3	320	160	48	360	360	51
18	r	NorESM1-M	144	96	26	320	160	53

[a] the fifith phase of the Coupled Model Intercmparison Project,
For further datails of models, see Table 9.A.1 in Flato et al. (2013).

Table S3 Features of 15 CMIP3[a] models

No	Label used in Figs S1-S3	Model name	Atmosphere			Ocean		
			Number of grids in longitude	Number of grids in latitude	Number of vertical levels	Number of grids in longitude	Number of grids in latitude	Number of vertical levels
1	a	BCCR-BCM2.0	128	64	31	240	120	35
2	b	CCSM3	256	128	26	360	180	40
3	c	CGCM3.1(T47)	96	48	31	189	94	29
4	d	CGCM3.1(T63)	128	64	31	400	128	29
5	e	CNRM-CM3	128	64	45	180	90	31
6	f	CSIRO-MK3.0	192	96	18	450	94	31
7	g	ECHAM5/MPI-OM	192	96	31	240	120	40
8	h	FGOALS-g1.0	128	60	26	360	180	16
9	i	GFDL-CM2.0	144	90	24	360	180	16
10	j	GISS-AOM	90	60	12	120	45	16
11	k	INM-CM3.0	72	45	21	180	72	33
12	l	MIROC3.2(hires)	320	160	56	1800	600	47
13	m	MIROC3.2(medres)	128	64	20	257	128	43
14	n	MRI-CGCM2.3.2	128	64	30	180	72	23
15	o	PCM	128	64	26	514	163	40

[a] the third phase of the Coupled Model Intercmparison Project,
For further datails of models, see Table 8.1 in Randall et al. (2007).

Table S4 Climate sensitivity[a] of CMIP5 models used in this study

No.	Model	Equilibrium Climate Sensitivity (°C)	Transient Climate Response (°C)	Tipping year global average		
				pav	*r5d*	*r1d*
1	ACCESS1.0	3.8	2.0	2047.4	2071.2	2070.0
2	CNRM–CM5	3.3	2.1	2054.4	2073.0	2073.5
3	INM–CM4	2.1	1.3	2054.0	2074.6	2075.6
4	IPSL–CM5A–LR	4.1	2.0	2043.9	2062.5	2060.1
5	IPSL–CM5B–LR	2.6	1.5	2057.1	2072.6	2071.2
6	MIROC5	2.7	1.5	2053.3	2072.0	2070.3
7	MIROC–ESM	4.7	2.2	2038.7	2062.1	2063.5
8	MPI–ESM–LR	3.6	2.0	2050.6	2069.0	2066.6
9	MRI–CGCM3	2.6	1.6	2056.2	2075.3	2075.4
10	NorESM1–M	2.8	1.4	2049.2	2072.3	2072.1

[a] The climate sensitivity of climate model is defined as the change in the global mean surface air temperature at the doubling of CO_2 concentraion. Out of 18 models used in this study, climate sensitivities of 10 models are only available from the Table 9.5 in (*11*). For technical detail, see (*11*).

Table S5 Data sources

Item	Website
Models data of the fifth phase of Coupled Model Intercomparison Project (CMIP5)	https://pcmdi.llnl.gov/mips/cmip5/
Models data of the third phase of Coupled Model Intercomparison Project (CMIP3)	https://pcmdi.llnl.gov/mips/cmip3/
Prtecipitation data of the Global Precipitation Climatology Project (GPCP)	https://precip.gsfc.nasa.gov/

References

Adler, R. F., G. J. Huffman, A. Chang, R. Ferrano, P.-P. Xie, J. Janowiak, B. Rudolf, U. Schneider, S. Curtis, D. Bolvin, A. Gruber, J. Susskind, P. Arkin and E. Nelkin, 2003: The Version−2 Global Precipitation Climatology Project (GPCP) monthly precipitation analysis (1979−Present). *J. Hydrometeor.*, **4**, 1147−1167, doi:10.1175/1525-7541(2003)004<1147:TVGPCP>2.0.CO;2.

Flato, G., J. Marotzke, B. Abiodun, P. Braconnot, S.C. Chou, W. Collins, P. Cox, F. Driouech, S. Emori, V. Eyring, C. Forest, P. Gleckler, E. Guilyardi, C. Jakob, V. Kattsov, C. Reason and M. Rummukainen, 2013: Chapter 9, Evaluation of Climate Models. In: Climate Change 2013: The Physical Science Basis. Contribution of Working Group I to the Fifth Assessment Report of the Intergovernmental Panel on Climate Change [Stocker, T.F., D. Qin, G.-K. Plattner, M. Tignor, S.K. Allen, J. Boschung, A. Nauels, Y. Xia, V. Bex and P.M. Midgley (eds.)]. Cambridge University Press, Cambridge, United Kingdom and New York, NY, USA.

Huffman, G. J., R. F. Adler, M. M. Morrissey, D. T. Bolvin, S. Curtis, R. Joyce, B. McGavock and J. Susskind, 2001: Global precipitation at one-degree daily resolution from multisatellite observations. *J. Hydrometeor.*, **2**, 36−50, doi:10.1175/1525-7541(2001)002<0036:GPAODD>2.0.CO;2.

Pfahl, S., P. A. O'Gorman, and E. M. Fischer, 2017: Understanding the regional pattern of projected future changes in extreme precipitation. *Nat. Clim. Chang.*, **7**, 423–427, doi:10.1038/nclimate3287.

Randall, D.A., R.A. Wood, S. Bony, R. Colman, T. Fichefet, J. Fyfe, V. Kattsov, A. Pitman, J. Shukla, J. Srinivasan, R.J. Stouffer, A. Sumi and K.E. Taylor, 2007: Chapter 8, Climate Models and Their Evaluation. In: Climate Change 2007: The Physical Science Basis. Contribution of Working Group I to the Fourth Assessment Report of the Intergovernmental Panel on Climate Change [Solomon, S., D. Qin, M. Manning, Z. Chen, M. Marquis, K.B. Averyt, M.Tignor and H.L. Miller (eds.)]. Cambridge University Press, Cambridge, United Kingdom and New York, NY, USA.

Simmons, A. J., A. Untch, C. Jakob, P. Kallberg, and P. Unden, 1999: Stratospheric water vapour and tropical tropopause temperatures in ECMWF analyses and multi-year simulations. Q. J. R. Meteorol. Soc., 125, 353-386, doi:10.1002/qj.49712555318.

Taylor, K. E., 2001: Summarizing multiple aspects of model performance in a single diagram. *J. Geophys. Res.*, **106**, 7183−7192, doi:10.1029/2000JD900719.
